# Flavin-containing monooxygenase 2 confers cardioprotection in ischemia models through its disulfide bond catalytic activity

**DOI:** 10.1172/JCI177077

**Published:** 2024-10-31

**Authors:** Qingnian Liu, Jiniu Huang, Hao Ding, Yue Tao, Jinliang Nan, Changchen Xiao, Yingchao Wang, Rongrong Wu, Cheng Ni, Zhiwei Zhong, Wei Zhu, Jinghai Chen, Chenyun Zhang, Xiao He, Danyang Xiong, Xinyang Hu, Jian’an Wang

**Affiliations:** 1 Department of Cardiology, The Second Affiliated Hospital, Zhejiang University School of Medicine, Hangzhou, Zhejiang, China.; 2 Cardiovascular Key Laboratory of Zhejiang Province, Hangzhou, Zhejiang, China.; 3 Institute of Translational Medicine, Zhejiang University School of Medicine, Hangzhou, Zhejiang, China.; 4 Shanghai Engineering Research Center of Molecular Therapeutics and New Drug Development, Shanghai Frontiers Science Center of Molecule Intelligent Syntheses, School of Chemistry and Molecular Engineering, East China Normal University, Shanghai, China.; 5 State Key Laboratory of Transvascular Implantation Devices, Hangzhou, Zhejiang, China.

**Keywords:** Cardiology, Cardiovascular disease, Cell stress, Chaperones

## Abstract

Myocardial infarction (MI) is characterized by massive cardiomyocyte (CM) death and cardiac dysfunction, and effective therapies to achieve cardioprotection are greatly needed. Here, we report that flavin-containing monooxygenase 2 (FMO2) levels were markedly increased in CMs in both ex vivo and in vivo models of ischemic injury. Genetic deletion of *FMO2* resulted in reduced CM survival and enhanced cardiac dysfunction, whereas CM-specific *FMO2* overexpression conferred a protective effect in infarcted rat hearts. Mechanistically, FMO2 inhibited the activation of ER stress–induced apoptotic proteins, including caspase 12 and C/EBP homologous protein (CHOP), by downregulating the unfolded protein response pathway. Furthermore, we identified FMO2 as a chaperone that catalyzes disulfide bond formation in unfolded and misfolded proteins through its GVSG motif. GVSG-mutated FMO2 failed to catalyze disulfide bond formation and lost its protection against ER stress and CM death. Finally, we demonstrated the protective effect of FMO2 in a human induced pluripotent stem cell–derived CM model. Collectively, this study highlights FMO2 as a key modulator of oxidative protein folding in CMs and underscores its therapeutic potential for treating ischemic heart disease.

## Abstract 2

FMO2 is associated with the prognosis of cardiac injury and may be an important therapeutic target for treating myocardial infarction and heart failure.

## Graphical abstract

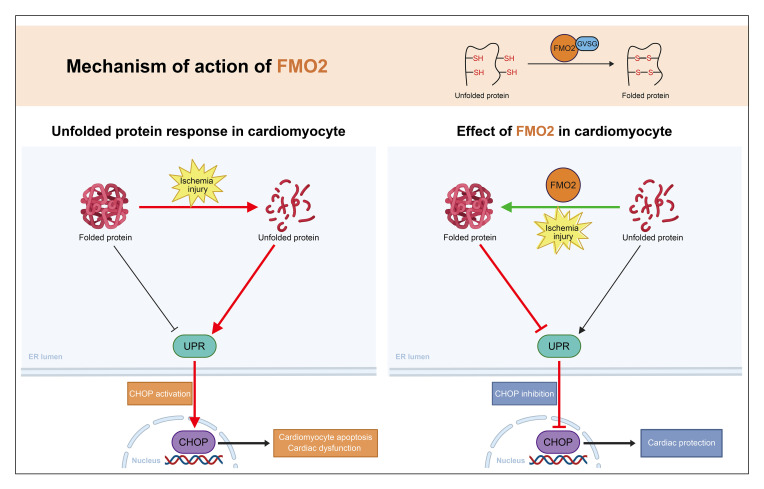

## Introduction

Myocardial infarction (MI) is the most severe manifestation of coronary heart disease that causes more than 7 million deaths per year (1). Even with advances of reperfusion strategies and pharmacological therapies, MI remains a serious threat to global health (1). Massive cardiomyocyte (CM) death during ischemia is recognized as the most important pathogenic process in MI-induced cardiac injury (2, 3), subsequently leading to the progression of heart failure (HF) (3–5). However, the fundamental mechanisms underlying CM death remain to be fully understood, and effective therapies are still lacking.

The reduction/oxidation (redox) reaction has close interaction with many physiological and pathological processes during MI. Upon oxygen deprivation, the initiated redox reaction acts as a regulator by changing protein conformation, stability, and activity to ensure basic signal transduction, gene transcription, cellular metabolism and ionic balance in CMs, whereas excessive redox reaction intensity damages the cells (6–8). Accumulating studies have uncovered important roles of proteins, such as heme oxygenase 1 (HO-1) (9), protein disulfide isomerase (PDI) (7, 10) and NADPH oxidase (Nox) (11, 12), which are involved in the redox process in modulating CM apoptosis and heart function. However, the mechanisms of the redox process in MI are still not well understood. We performed RNA-Seq analysis in hypoxia neonatal rat CMs (NRCMs) and identified flavin-containing monooxygenase 2 (*FMO2*) as one of the most markedly induced genes closely related to the redox process. FMOs (FMO1–5) are located in the ER (13, 14) and catalyze the oxidative reaction to foreign chemicals such as imipramine, aldicarb, promethazine, etc. (15). FMO2 requires molecular oxygen for oxidation, with flavin adenine dinucleotide (FAD) and nicotinamide adenine dinucleotide phosphate (NADPH) as cofactors (13, 16). Recently, we discovered that FMO2 expression was downregulated in fibroblasts and exhibited enzyme-independent antifibrotic activity in infarcted hearts, while expression of FMO2 in CMs was elevated (17), which prompted us to ask whether FMO2 also plays a crucial role in CMs in the ischemic heart.

In the current study, we found that overexpression of FMO2 both in vivo and in vitro was sufficient to reduce MI-induced CM apoptosis and preserve cardiac function. This cardioprotective activity was mediated by modulating the unfolded protein response (UPR) pathway and subsequently attenuating the activation of downstream C/EBP homologous protein (CHOP) activation and caspase 12 cleavage. Notably, we identified FMO2 as an ER chaperone with GVSG-dependent disulfide bond catalytic activity in CMs. In addition, the translational relevance of our finding was supported by the results of the FMO2-mediated protective effect in human induced pluripotent stem cell–derived CMs (hiPSC-CMs). Thus, our study reveals the critical role of FMO2 in facilitating oxidative protein folding in the ER and underscores its therapeutic potential for MI treatment.

## Results

### FMO2 is induced in CMs in a post-MI model in vivo and hypoxia culture in vitro.

**Figure 1:**
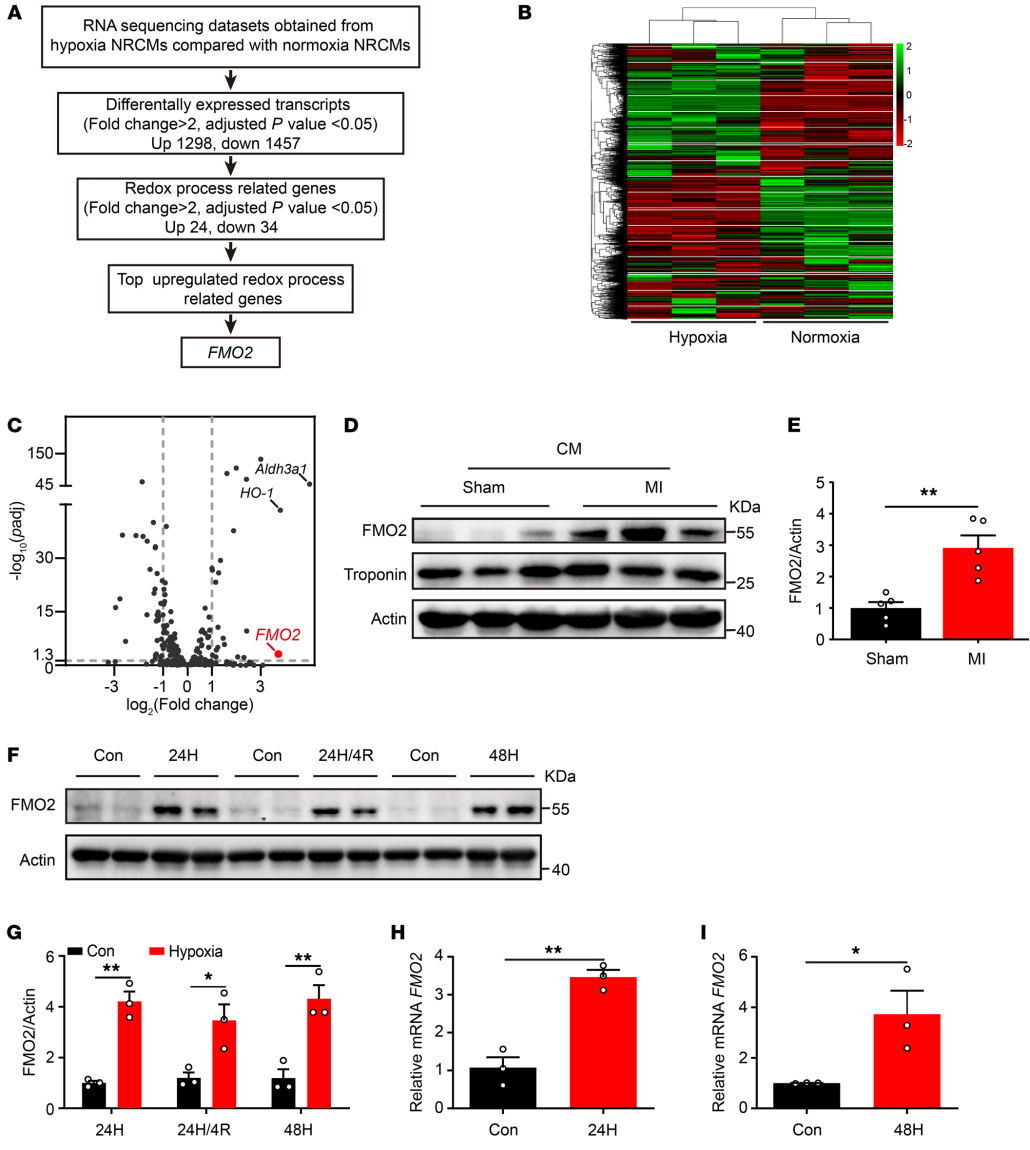
FMO2 expression is increased in CMs upon ischemic injury. (**A**) Workflow of the discovery of FMO2 in NRCMs subjected to hypoxia for 24 hours. (**B**) Hierarchical clustering of differentially expressed genes in hypoxic NRCMs compared with normoxic NRCMs as indicated (fold change >2 or <0.5, adjusted *P* < 0.05). (**C**) Volcano map showed differentially expressed genes involved in the oxidation reduction process in hypoxic NRCMs compared with normoxic NRCMs (fold change >2 or <0.5, adjusted *P* < 0.05). (**D** and **E**) Western blotting was performed to measure the expression of FMO2 in CMs isolated from normal and infarcted adult rat hearts (*n* = 5 per group). (**F** and **G**) FMO2 protein expression in NRCMs under different hypoxia or H/R conditions (*n* = 3 independent experiments). 24H, 24 hours of hypoxia; 24H/4R, 24 hours of hypoxia/4 hours of reoxygenation; 48H, 48 hours of hypoxia. (**H** and **I**) *FMO2* mRNA expression in NRCMs subjected to hypoxia for 24 or 48 hours (*n* = 3 independent experiments). Con, control. Data are presented as the mean ± SEM, and significance was evaluated by 2-tailed Student’s *t* test. *P < 0.05 and **P < 0.01.

To explore the underlying mechanisms of the redox process in CM apoptosis, we performed RNA-Seq analysis in normoxic NRCMs and hypoxic NRCMs (Figure 1A). A total of 2,755 differentially expressed transcripts (fold change >2 or <0.5, adjusted *P* < 0.05) were identified in hypoxic NRCMs compared with the normoxic control (Figure 1B and Supplemental Figure 1A; supplemental material available online with this article; https://doi.org/10.1172/JCI177077DS1). Gene Ontology (GO) enrichment analysis revealed that the oxidation reduction process was one of the top enriched pathways under hypoxia (Supplemental Figure 1B). Among these transcripts involved in the oxidation reduction process, *FMO2*, aldehyde dehydrogenase 3 family member A1 (*Aldh3a1*) and *HO-1* were the most highly expressed (Figure 1C), yet the role of FMO2 in CM injury has not been reported, to our knowledge. A notable increase in FMO2 protein expression was observed in CMs from adult rat hearts isolated 3 days after MI compared with sham-operated samples (Figure 1, D and E, and Supplemental Figure 1, C and D). In cultured NRCMs, the protein and mRNA levels of FMO2 were also markedly increased after hypoxia treatment for 24 or 48 hours (Figure 1, F–I), as well as after hypoxia/reoxygenation (H/R) treatment (Supplemental Figure 1, E and F). Similar to FMO2, FMO1 and FMO4 protein levels were modestly elevated in NRCMs subjected to hypoxia for 24 hours (Supplemental Figure 1, G, H, K, and L), while FMO3 and FMO5 protein levels were not affected (Supplemental Figure 1, I, J, M, and N). We detected extremely low expression levels of FMO3 in CMs. These findings suggest that FMO2 expression was dramatically increased in hypoxic CMs, yet its role in response to ischemic injury is unknown.

Next, we investigated the expression and distribution of FMO2 in heart samples from patients with MI and non-MI controls. In normal hearts, FMO2 expression in CMs was modest, however, in MI hearts, its expression was markedly increased (Supplemental Figure 2A). Specifically, MI led to a marked increase in FMO2 expression in CMs, whereas its expression in fibroblasts decreased (Supplemental Figure 2A). Our findings revealed that, in MI patients, FMO2 expression in CMs far exceeded that in fibroblasts. In addition, we validated our findings using a hiPSC-CM model. hiPSC-CMs were differentiated from hiPSCs as previously described (18) and characterized by the expression of the markers troponin I and α-sarcomeric actinin (α-actinin) (Supplemental Figure 2B). Flow cytometric analysis showed that 95.7% of hiPSC-derived cells expressed troponin T, confirming their identity as hiPSC-CMs (Supplemental Figure 2C). Following hypoxia exposure, FMO2 expression in hiPSC-CMs dramatically increased (Supplemental Figure 2, D and E), further supporting our observations. In addition to evaluating FMO2 expression in the NRCM and hiPSC-CM models, we also examined its changes in neonatal rat cardiac fibroblasts (NRCFs). After hypoxia exposure, HIF1 protein expression increased in NRCF cells, while expression of FMO2 decreased (Supplemental Figure 2, F and G), consistent with our findings in MI patients’ hearts.

### CM-specific FMO2 knockdown increases CM apoptosis and deteriorates heart function.

**Figure 2:**
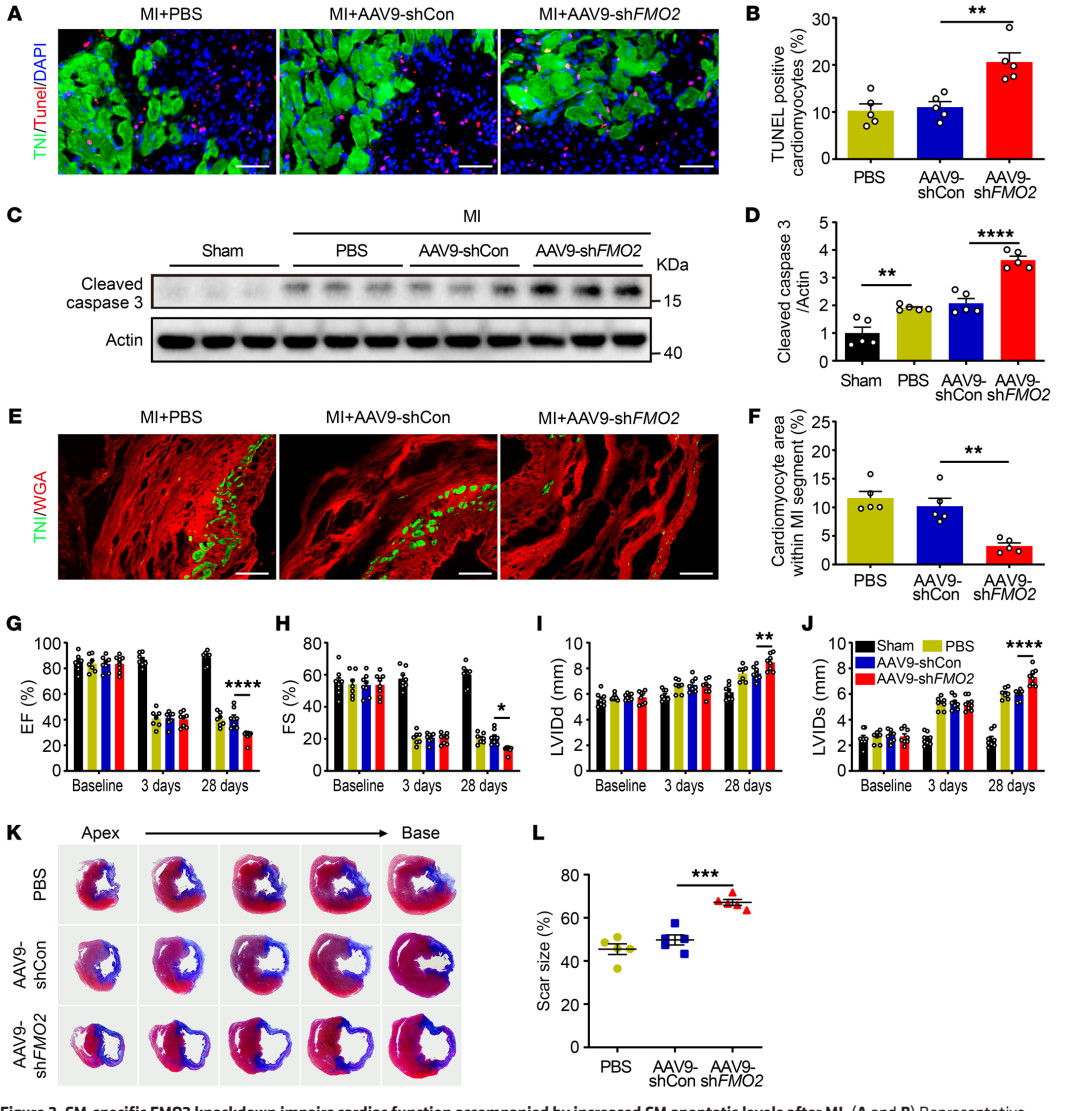
CM-specific FMO2 knockdown impairs cardiac function accompanied by increased CM apoptotic levels after MI. (**A** and **B**) Representative images (**A**) of sections of rat hearts subjected to MI were analyzed for apoptosis by TUNEL staining in the border area. Scale bar: 50 μm. Quantitative analysis (**B**) of TUNEL^+^ and troponin I^+^ CMs (*n* = 5 per group). (**C** and **D**) Western blots (**C**) of protein expression of cleaved caspase 3 in rats that either received AAV9-sh*FMO2* or AAV9-shCon virus and (**D**) quantification of the protein lysates harvested from the infarct border zone of hearts (*n* = 5 per group). (**E** and **F**) Images (**E**) of the scar area stained with WGA (red) and troponin I (green) (scale bars: 100 μm) and (**F**) quantitative analysis of the troponin I^+^ area proportion (*n* = 5 per group). (**G**–**J**) Quantitative analysis of echocardiographic results (*n* = 7–9 per group). (**K**) Representative tissue sections stained with Masson’s at post-MI day 28. (**L**) Scar size percentage (*n* = 5 per group). Data are presented as the mean ± SEM. Comparisons among 3 or more groups were evaluated by 1-way ANOVA with Tukey’s test, and comparisons among groups after multiple treatments were evaluated by 2-way ANOVA with Tukey’s test. *P < 0.05, **P < 0.01, ***P < 0.001, and ****P < 0.0001.

To test the role of FMO2 in response to ischemic injury, Sprague-Dawley (SD) rats received a single intravenous tail vein injection (200 μL) of an adenovirus-associated virus serotype 9 (AAV9) vector harboring *FMO2* shRNA (AAV9-sh*FMO2*, 4 × 10^12^ viral particles per rat) or a nonspecific shRNA (AAV9-shCon). We used an AAV9 vector in which the cardiac-specific troponin T promoter (cTnT) had been incorporated to achieve CM-specific FMO2 knockdown. Four weeks after AAV injection, the rats were subjected to MI surgery. AAV9-sh*FMO2* dramatically decreased the protein and mRNA levels of FMO2 in rat CMs (Supplemental Figure 3, A–C). Three days after MI, quantification of apoptotic levels in the infarct border area indicated higher levels of TUNEL–troponin I (TnI) double-positive CMs (Figure 2, A and B) and higher levels of cleaved caspase 3 (Figure 2, C and D) in the AAV9-sh*FMO2*–treated hearts than in the AAV9-shCon–treated hearts. Also, we detected fewer viable CMs in the MI segments of AAV9-sh*FMO2* hearts 28 days after MI (Figure 2, E and F). Cardiac function was decreased in the AAV9-sh*FMO2*–treated rats (Figure 2, G–J, and Supplemental Figure 3D), as evidenced by the decreased ejection fraction (EF), dilated ventricular chamber, and enlarged scar size (Figure 2, K and L). We also evaluated fibrosis in the AAV9-sh*FMO2* group that did not undergo surgery and found no significant changes compared with the control (Supplemental Figure 3, E and F). Next, we generated a *FMO2*-KO (*FMO2^–/–^*) rat by CRISPR/Cas9-mediated genome editing (Supplemental Figure 4C). Consistent with the results from FMO2 knockdown, we detected more TUNEL-TnI double-positive CMs in the infarct border zone of *FMO2^–/–^* hearts (Supplemental Figure 4, A and B) at post-MI day 3, along with increased levels of cleaved caspase 3 protein expression (Supplemental Figure 4, C and D). *FMO2^–/–^* rats exhibited deteriorated cardiac function as measured by echocardiography on day 28 after MI injury compared with WT rats (Supplemental Figure 4, E–I). All of these observations indicate that FMO2 deficiency in CMs deteriorated cardiac dysfunction after MI.

### CM-specific FMO2 overexpression inhibits CM apoptosis and improves cardiac function.

**Figure 3:**
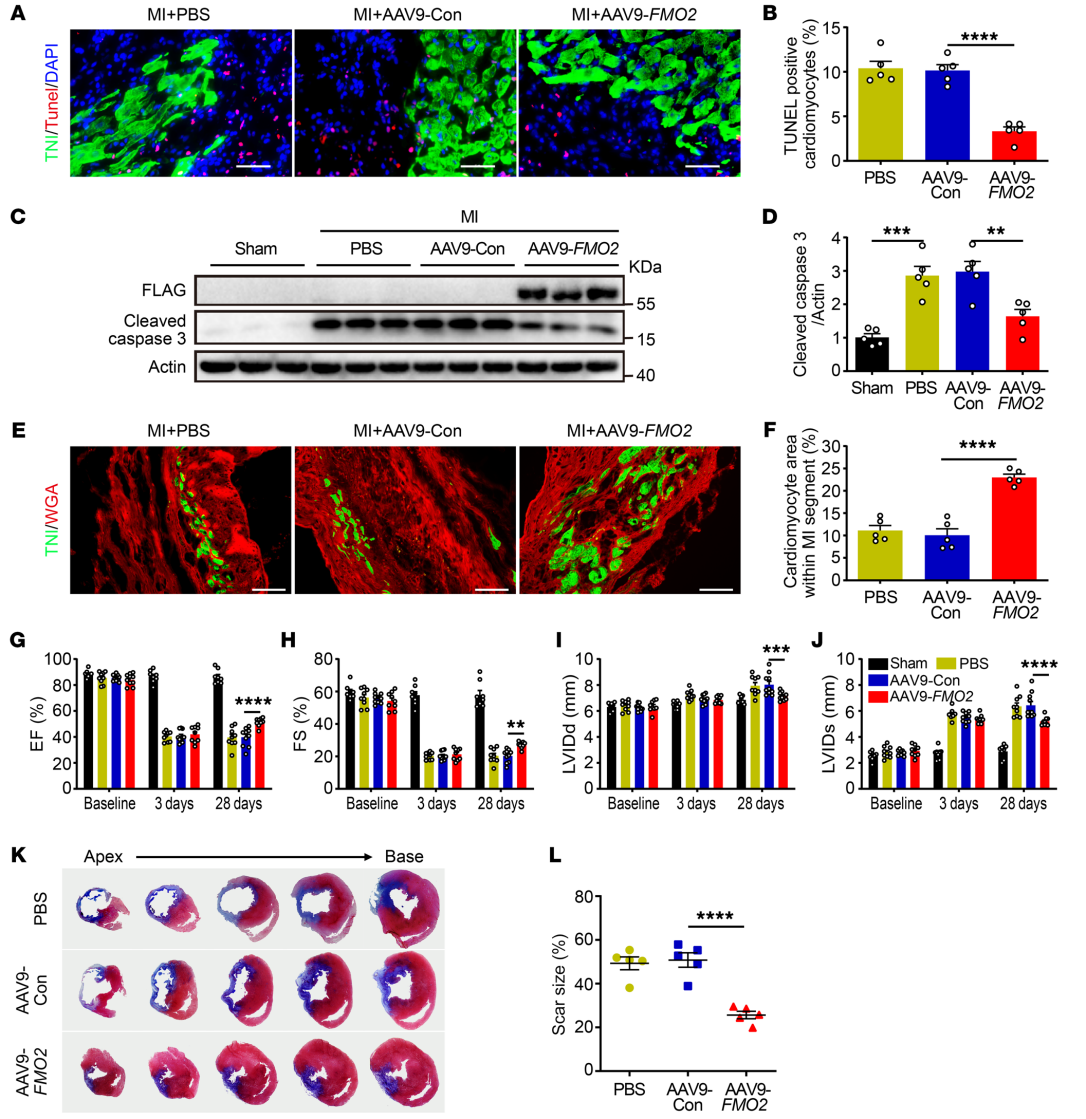
CM-specific FMO2 overexpression decreases CM apoptosis and improves cardiac function after MI. (**A** and **B**) Images (**A**) and analysis (**B**) of TUNEL^+^ and troponin I^+^ CMs in the border zone of infarcted hearts (*n* = 5 per group). Scale bars: 50 μm. (**C** and **D**) Western blots (**C**) and analysis (**D**) of protein expression of FMO2-FLAG and cleaved caspase 3 in rats that received either AAV9-*FMO2* or AAV9-Con virus (*n* = 5 per group). (**E** and **F**) Images (**E**) of the scar area stained with WAG (red) and troponin I (green) (scale bars: 100 μm) and (**F**) quantitative analysis of the troponin I^+^ area proportion is shown in (right, *n* = 5 per group). (**G**–**J**) EF, FS, LVIDd, and LVIDs were measured by echocardiography (*n* = 8 in the sham group, *n* = 9–10 in the other groups). (**K** and **L**) Representative images (**K**) of tissue sections stained with Masson on post-MI day 28 and (**L**) the scar size percentage (*n* = 5 per group). Data are presented as the mean ± SEM. Comparisons among 3 or more groups were evaluated by 1-way ANOVA with Tukey’s test, and comparisons among groups after multiple treatments were evaluated by 2-way ANOVA with Tukey’s test. **P < 0.01, ***P < 0.001, and ****P < 0.0001.

To achieve CM-specific overexpression of FMO2, rats were injected with the AAV9-cTnT vector encoding the rat *FMO2* gene (AAV9-*FMO2*, a total of 4 × 10^12^ viral particles per rat) or a negative control AAV9-luciferase 2 (AAV9-Con) and subjected to MI surgery 4 weeks later. Compared with AAV9-Con, AAV9-*FMO2*–injected hearts showed high levels of FMO2-FLAG protein (Supplemental Figure 5A) and mRNA expression (Supplemental Figure 5B). Three days after MI, the TUNEL-TnI double-positive CMs in the infarct border area of AAV9-*FMO2* rats were reduced in number (Figure 3, A and B), and cleaved caspase 3 protein levels were markedly decreased compared with those in AAV9-Con rats (Figure 3, C and D). In addition, rats in the AAV9-*FMO2* group showed more viable CMs in the MI segments at post-MI day 28 (Figure 3, E and F), along with improved cardiac function, as measured by echocardiography (Figure 3, G–J, and Supplemental Figure 5C), and reduced scar size (Figure 3, K and L). These results further support notion that FMO2 has a critical role in cardiac protection after MI injury.

### FMO2-mediated CM protection is dependent on enzymatic activity.

**Figure 4:**
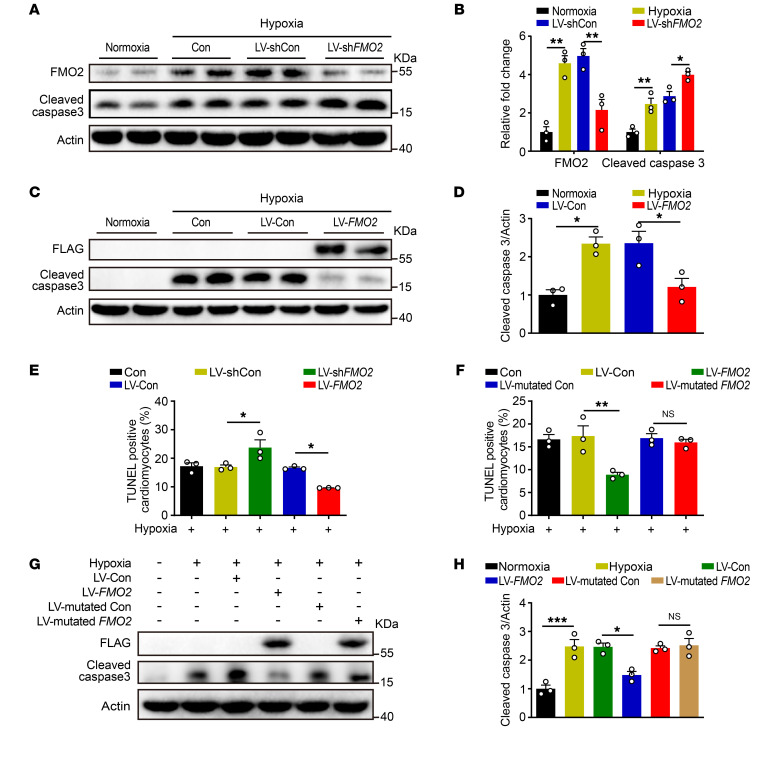
FMO2 confers protection from hypoxia-induced apoptosis via its enzymatic activity in cultured CMs. (**A** and **B**) Western blots (**A**) and analysis (**B**) of protein expression of cleaved caspase 3 and FMO2 in NRCMs transfected with LV-sh*FMO2* or LV-shCon, and then subjected to hypoxia. (**C** and **D**) Western blots (**C**) and analysis (**D**) of protein expression of cleaved caspase 3 in hypoxia-exposed NRCMs with LV-*FMO2*-FLAG or LV-Con. (**E**) Quantitative analysis of NRCMs that were transfected with either LV-sh*FMO2* or LV-*FMO2* lentivirus and then subjected to hypoxia for 24 hours. (**F**) Quantitative analysis of TUNEL staining of hypoxia-exposed NRCMs with LV-*FMO2* or enzyme-inactivated *FMO2* (LV-mutated *FMO2*). (**G** and **H**) Western blots (**G**) and analysis (**H**) of cleaved caspase 3 protein levels in hypoxia-exposed NRCMs with LV-*FMO2*-FLAG or LV-mutated *FMO2*-FLAG. The graphs summarize data from 3 independent experiments. Data are presented as the mean ± SEM, and significance was evaluated by 1-way ANOVA with Tukey’s test among 3 or more groups. *P < 0.05, **P < 0.01, and ***P < 0.001.

In order to investigate the effect of FMO2 on CM apoptosis in vitro, we isolated NRCMs and transfected them with lentiviruses containing either a *FMO2*-targeted shRNA (LV-sh*FMO2*) or *FMO2* cDNA (LV-*FMO2*), while a nonspecific shRNA (LV-shCon) or an empty lentiviral plasmid (LV-Con) served as the control. Following this treatment, we subjected NRCMs to hypoxia for 24 hours. Loss of FMO2 enhanced NRCM apoptosis, while FMO2 overexpression reduced cell apoptosis as measured by cleaved caspase 3 protein level (Figure 4, A–D) and TUNEL staining (Figure 4E and Supplemental Figure 6A).

To further determine whether FMO2-mediated cardioprotection is dependent on its enzymatic activity, we tested the effect of an enzyme-inactivated FMO2 carrying mutations at its FAD and NADPH binding sites using a lentivirus (LV-mutated *FMO2*) (17). As shown in Figure 4, F–H, and Supplemental Figure 6B, overexpression of this mutant FMO2 in NRVMs failed to confer any notable protection against hypoxia-induced apoptosis. These results suggest that the cardioprotective effect of FMO2 was dependent on its enzymatic activity.

### FMO2 ameliorates apoptotic response through the UPR pathway.

**Figure 5:**
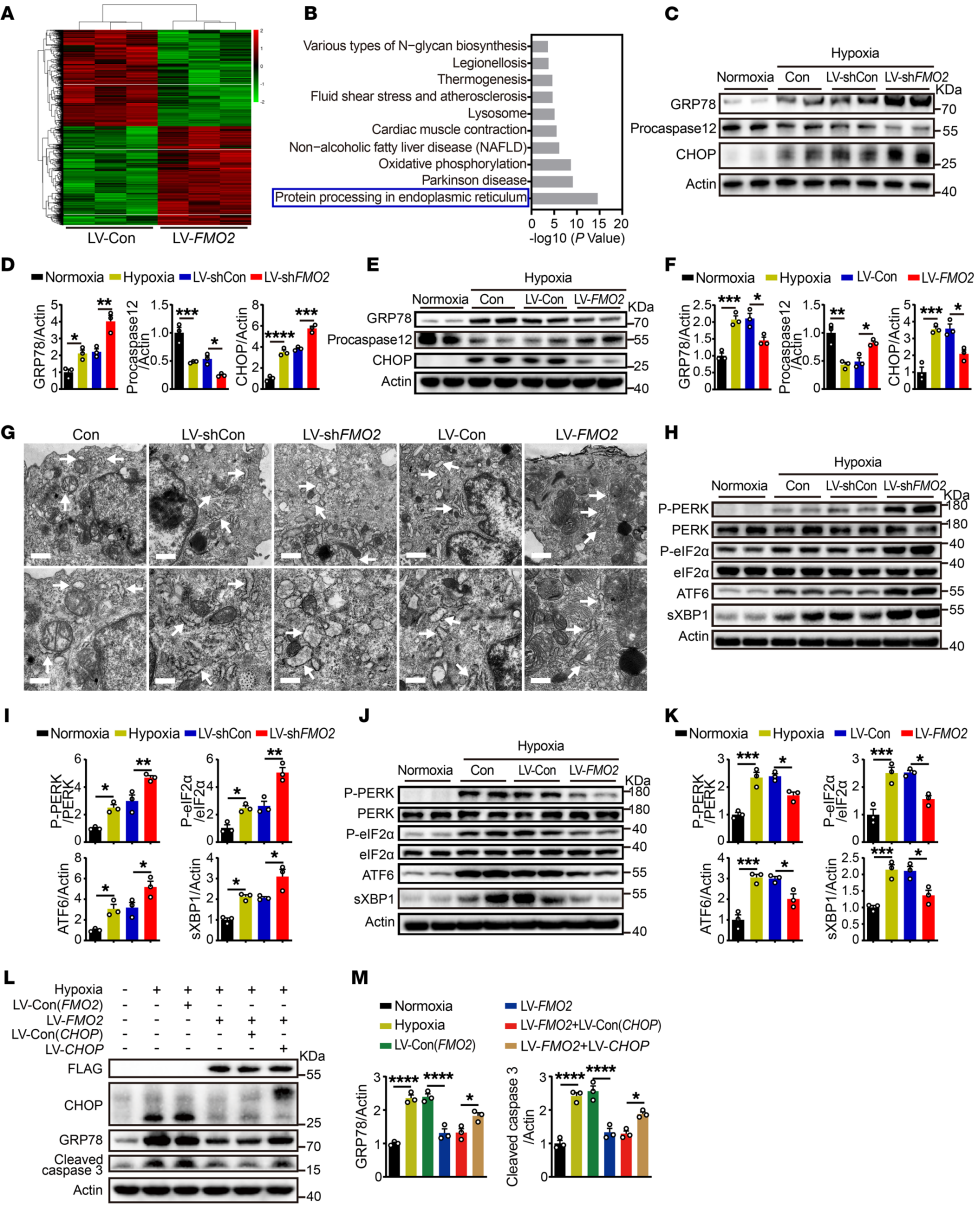
The apoptotic response is ameliorated by FMO2 through the UPR pathway in cultured CMs. (**A**) Hierarchical clustering of differentially expressed genes in hypoxia-exposed NRCMs with LV-*FMO2* or LV-Con (fold change >2 or <0.5, adjusted *P* < 0.05). (**B)** Pathway enrichment analysis of markedly altered genes identified between LV-*FMO2* and LV-Con NRCMs. (**C**–**F**) Protein expression of GRP78 and ER stress–induced apoptotic proteins including procaspase 12 and CHOP in hypoxia-exposed NRCMs with LV-sh*FMO2* or LV-*FMO2* lentivirus. (**G**) ER morphology in each group of NRCMs was observed by TEM. Scale bars: 1 μm (top) and 0.5 μm (bottom). The images below are higher magnifications of the ones above. Arrows indicate the ER. (**H**–**K**) Alterations of UPR signaling in hypoxia-exposed NRCMs with LV-sh*FMO2* or LV-*FMO2*. (**L** and **M**) Protein expression of ER stress–associated proteins in hypoxia-exposed NRCMs with *FMO2*-FLAG and *CHOP* overexpression lentiviruses. The graphs summarize data from 3 independent experiments. Data are presented as the mean ± SEM, and significance was evaluated by 1-way ANOVA with Tukey’s test among 3 or more groups. *P < 0.05, **P < 0.01, ***P < 0.001, and ****P < 0.0001. GRP78, glucose-regulated protein 78 kDa.

FMO2 is an ER transmembrane–targeted protein. However, its role is mostly implicated in the detoxification of foreign chemicals through oxygenase activity. To better understand the molecular mechanisms underlying FMO2-mediated cardiac protection, we performed RNA-Seq analysis using FMO2-overexpressing CMs subjected to hypoxia for 24 hours. Compared with the control, a total of 990 differentially expressed transcripts (fold change >2 or <0.5, adjusted *P* < 0.05) were identified, 709 of which were upregulated and 281 were downregulated (Figure 5A). Among the top 10 Kyoto Encyclopedia of Genes and Genomes (KEGG) enrichment pathways, the most relevant one was for protein processing in the ER pathway (Figure 5B). Cardiac ischemia impairs ER function, thereby causing the accumulation of improperly folded or unfolded proteins in the lumen, which is referred to as ER stress (19). Upon ER stress, the UPR pathway including the 3 interactive signaling branches protein kinase RNA-like ER kinase/eukaryotic initiation factor 2 α subunit (PERK/eIF2α), spliced X-box–binding protein 1 (sXBP1), and activating transcription factor 6 (ATF6) are activated to resolve the toxic unfolded proteins (19, 20). Nevertheless, if ER stress is severe or persistent, specific ER stress–induced apoptotic proteins are triggered, including caspase 12 and CHOP, leading to apoptosis of CMs and eventually the deterioration of heart attack (19, 21). Consistent with the RNA-Seq data, we found that FMO2 knockdown increased ER stress, resulting in upregulated protein expression of glucose-regulated protein 78kDa (GRP78), CHOP, and cleaved caspase 12 (Figure 5, C and D), whereas FMO2 overexpression had the opposite effect (Figure 5, E and F). Electron microscopy showed that FMO2 downregulation increased ER volume in hypoxia-treated NRCMs, while FMO2 overexpression ameliorated this change (Figure 5G). Indeed, we found that expression levels of phosphorylated PERK (p-PERK), p-eIF2α, sXBP1, and ATF6 were all upregulated by FMO2 knockdown in NRCMs under hypoxia (Figure 5, H and I). Meanwhile, overexpression of FMO2 markedly attenuated ER stress pathways, as demonstrated by the reduction of p-PERK/PERK, p-eIF2α/eIF2α, sXBP1, and ATF6 levels (Figure 5, J and K). Moreover, we found that CHOP overexpression impaired the FMO2-mediated protection against ER stress and ER stress–induced apoptosis (Figure 5, L and M). These results support the idea that FMO2 may reduce CM apoptosis through the UPR pathway.

**Figure 6:**
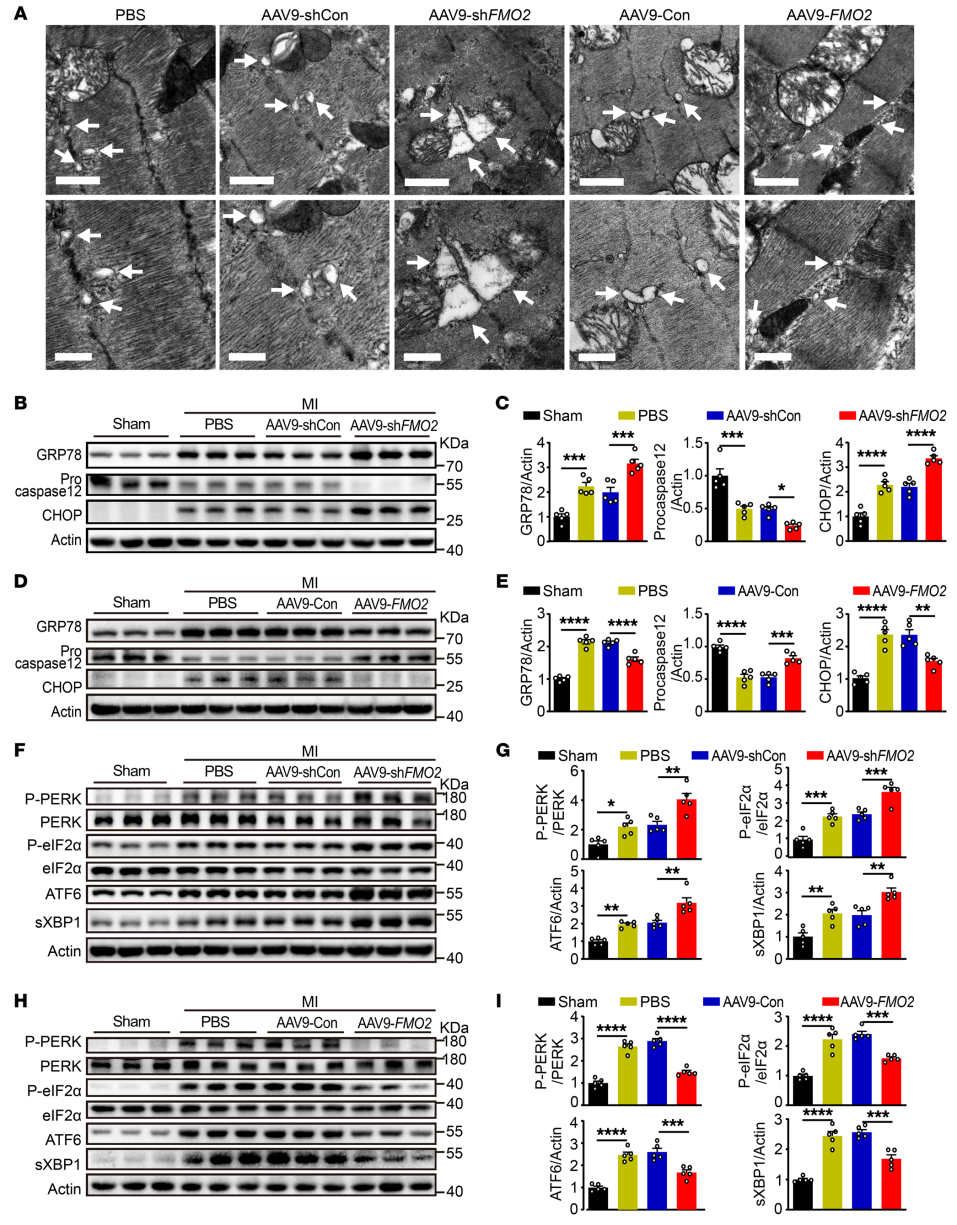
FMO2 exhibits cardiac-protective effects against apoptosis through the UPR pathway in infarcted hearts. (**A**) Electron microscopy analysis of infarcted rat hearts after administration of AAV9-sh*FMO2* or AAV9-*FMO2*. Scale bars: 1 μm (top) and 0.5 μm (bottom). The images below are higher magnifications of the ones above. Arrows indicate the ER. (**B**–**E**) Protein expression of GRP78, procaspase 12, and CHOP in infarcted hearts injected with AAV9-sh*FMO2* or AAV9-*FMO2*. (**F**–**I**) Alterations of UPR signaling in infarcted hearts with AAV9-sh*FMO2* or AAV9-*FMO2*. The graphs summarize data from 5 rats per group. Quantified data are presented as the mean ± SEM, and significance was evaluated by 1-way ANOVA with Tukey’s test among 3 or more groups. *P < 0.05, **P < 0.01, ***P < 0.001, and ****P < 0.0001.

To test the effect of FMO2 expression on ER stress in vivo, we isolated heart tissue from rats treated with AAV9-sh*FMO2* or AAV9-*FMO2* at post-MI day 3. Electron microscopy showed that AAV9-sh*FMO2* treatment exacerbated the dilation of the ER lumen compared with AAV9-Con, whereas FMO2 overexpression attenuated the dilation (Figure 6A). Consistent with the in vitro data, ER stress–induced apoptosis (as measured by caspase 12 cleavage and CHOP expression) and GRP78 expression were enhanced in the AAV9-sh*FMO2* hearts (Figure 6, B and C) and were accompanied by elevated levels of UPR stress signaling proteins, including p-PERK/PERK, p-eIF2α/eIF2α, sXBP1, and ATF6 (Figure 6, F and G). Conversely, FMO2 overexpression inhibited the ER stress–induced apoptotic response (Figure 6, D and E), as well as p-PERK/PERK, p-eIF2α/eIF2α, sXBP1, and ATF6 levels (Figure 6, H and I). Consistent with the results observed with AAV9-sh*FMO2* treatment, *FMO2^–/–^* rats exhibited enhanced ER stress and ER stress–induced apoptosis at post-MI day 3, as demonstrated by increased expression of GRP78, CHOP, cleaved caspase 12, and cleaved caspase 3 compared with WT rats (Supplemental Figure 7, A and B). Taken together, FMO2 expression ameliorated ER stress in vivo, which was associated with inhibition of the UPR and specific apoptotic response.

### FMO2-mediated cardiac protection against ER stress depends on its disulfide bond catalytic activity.

**Figure 7:**
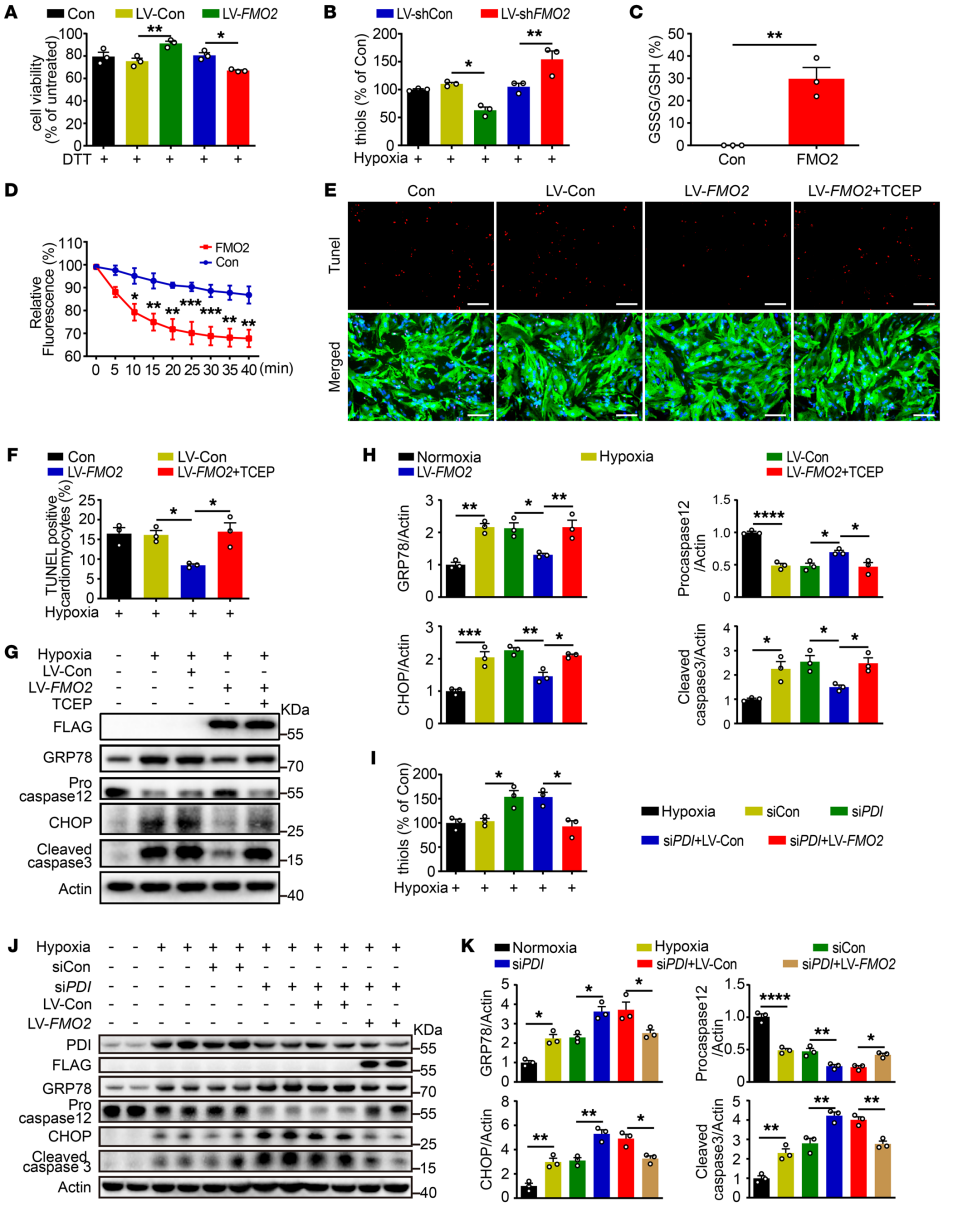
FMO2-mediated cardiac protection against ER stress is dependent on catalysis of the disulfide bond. (**A**) Viability of differently treated CMs was detected with the Cell Counting Kit-8 (CCK-8), normalized to a parallel culture of the same cells that had not been exposed to DTT. (**B**) Contents of reduced thiols in hypoxia-exposed NRCMs transfected with LV-sh*FMO2* or LV-*FMO2* lentivirus. Data shown for the differently treated groups were normalized to the hypoxia control. (**C** and **D**) The reaction of substrate GSH or peptide NRCSQGSCWN with FMO2. The GSSG/GSH ratio was measured, while the peptides was monitored according to fluorescence intensity (excitation 280 nm, emission 350 nm). (**E** and **F**) Images and analysis of TUNEL staining of hypoxia-exposed NRCMs treated or not with the disulfide bond–specific inhibitor TCEP. Scale bars: 100 μm. (**G** and **H**) Western blots and analysis showing alterations of GRP78 and ER stress–induced apoptotic proteins in hypoxia-exposed NRCMs with or without TCEP. (**I**) Contents of reduced thiols in hypoxia-exposed NRCMs transfected with *PDI* siRNA or LV-*FMO2* lentivirus. Bar diagram of differently treated groups was normalized to the hypoxia control. (**J** and **K**) Western blots and analysis of protein expression of GRP78 and ER stress–induced apoptotic proteins in hypoxia-exposed NRCMs with *PDI* siRNA and LV-*FMO2*-FLAG lentivirus. Graphs summarize data from 3 independent experiments. Data are presented as the mean ± SEM. Comparisons between 2 groups were assessed by 2-tailed Student’s *t* test, comparisons among 3 or more groups were evaluated by 1-way ANOVA with Tukey’s test, and comparisons among groups after multiple treatments were evaluated by 2-way ANOVA with Tukey’s test. *P < 0.05, **P < 0.01, ***P < 0.001, and ****P < 0.0001.

Canonical ER stress induction is triggered by improperly folded or unfolded proteins in the ER lumen. More than 30% of all human proteins are synthesized in the ER, the majority of which require disulfide bonds during maturation (10, 22). Recent studies have demonstrated that PDI is one of the most important enzymes in the catalysis of disulfide bond synthesis to facilitate oxidative protein folding in the ER (23, 24). Indeed, PDI-mediated disulfide bond synthesis needs the aid of an ER-associated oxidase named ER oxidoreductase 1 (ERO-1) (22). Like FMO2, ERO-1 requires FAD as a cofactor to complete multiple oxidation reactions (10). ERO-1 oxidizes reduced PDI to become functional oxidized PDI, thereby enabling PDI to catalyze disulfide bond formation in unfolded proteins (22). However, little is known about the role of FMO2 and the underlying mechanisms of disulfide bond synthesis. To explore whether FMO2 has a similar function, we examined the sensitivity of NRCMs to exogenously added DTT, a disulfide bond–reducing agent (25, 26). Interestingly, FMO2 expression conferred notable resistance against DTT-induced cytotoxicity, whereas FMO2 knockdown lost the effect (Figure 7A). Using 5,5′-dithiobis-(2-nitrobenzoic acid) (DTNB), we found the levels of thiols were lower in FMO2-overexpressing NRCMs, but higher in FMO2-knockdown cells compared with the control (Figure 7B). These results, together with the protective effect of FMO2 on ER stress, indicated that FMO2 might promote disulfide bond formation in CMs. Next, we explored whether FMO2-mediated regulation of the disulfide bond was carried out through PDI. FMO2 expression had no effect on PDI expression at either the protein or mRNA level (Supplemental Figure 8, A–C). In addition, we found no changes in the electrophoretic mobility of PDI in NRCMs after treatment with LV-*FMO2* or LV-sh*FMO2* using a 4-acetamido-4′-maleimidylstilbene-2,2′-disulfonic acid (AMS) modification assay (Supplemental Figure 8D), indicating that FMO2 promoted disulfide bond formation without redox modulation of PDI. Therefore, we proposed a hypothesis that, similar to PDI, FMO2 catalyzes disulfide bond synthesis directly. Mixing purified FMO2 protein (Supplemental Figure 9A) with glutathione (GSH) in test tubes, we found that thiols in GSH were oxidized to disulfide bonds in glutathione disulfide (GSSG) by FMO2 (Figure 7C and Supplemental Figure 9, B and C). Additionally, to directly monitor disulfide bond formation, we used a convincing method that utilized a decapeptide NRCSQGSCWN containing only 2 cysteine residues (Supplemental Figure 9D) (27–30). A tryptophan adjacent to 1 cysteine residue was used as a fluorophore, and an arginine adjacent to the other cysteine was designed as the charged quencher. If the cysteine residues on the peptide are not oxidized to a disulfide bond or are oxidized to other chemical groups such as sulfoxide (S-O) or sulfinic acid (S-OH), fluorescence quenching will not result. Intramolecular disulfide bond synthesis catalyzed by FMO2 could be directly observed by the quenching of tryptophan fluorophore (Figure 7D). Taken together, these results clearly demonstrate that FMO2 was able to catalyze disulfide bond synthesis.

To further investigate the relationship between ER stress and the disulfide bond catalytic activity of FMO2, we used a selective disulfide bond inhibitor, tris (2-carboxyethyl) phosphine (TCEP), to block disulfide bond formation in cells (31, 32). The induction of disulfide bond synthesis by FMO2 was abolished when NRCMs were treated with TCEP at 0.5 mM (Supplemental Figure 9E). In the meantime, the protective effect of FMO2 on ER stress and ER stress–induced apoptosis was blocked by TCEP treatment (Figure 7, E–H). Thus, FMO2-mediated cytoprotection against ER stress was dependent on its disulfide bond catalytic activity.

Next, we used siRNA to knock down PDI expression in NRCMs, which inhibited disulfide bond formation, and found that FMO2 overexpression could compensate for it (Figure 7I). PDI knockdown deteriorated ER stress and ER stress–induced apoptosis, but was rescued by FMO2 overexpression (Figure 7, J and K). These results suggest that increased FMO2 expression under hypoxic injury can compensate for the adverse effects of PDI deficiency on the folding of disulfide bond–containing proteins and ER stress.

### FMO2 depends on its GVSG motif for disulfide bond formation.

Considering the similar effect of FMO2- and PDI-mediated disulfide bond formation and the fact that FMO2 could counteract the detrimental effects of PDI deficiency on disulfide bond formation to relieve ER stress, we conducted a comparative structural analysis of these 2 proteins. PDI contains four thioredoxin-like domains arranged in the order of a-b-b′-a′ in a horseshoe shape (Supplemental Figure 10A). The structures of domain a and domain a′ were highly similar, with superposition a root mean square deviation (RMSD) score of just 1.55 (Supplemental Figure 10B). The CGHC motif in both domains is the active site responsible for disulfide bond formation in unfolded proteins and peptides (33). We utilized the crystalline structure of FMO2 (Protein Data Bank [PDB] ID: 6SF0), which yielded a complete structure containing both FAD and NADP+ (Supplemental Figure 10, C and D). FMO2 consisted of domains 1 and 2. After superposing the structure of PDI domain a/a′ with that of FMO2, the overlapped region was found in the thioredoxin-like domain (residues 1–33, 134–147, and 379–396) of FMO2 domain 1. The same secondary structure was β-strand–α-helix–β-strand (β1-α1-β2), β-strand–β-strand (β5-β6), and α-helix (α3), respectively (Supplemental Figure 10, C and D). However, a notable difference was identified in a motif containing residues 104–133 in FMO2, which showed a β-strand–α-helix–β-strand (β3-α2-β4) instead of an α-helix in PDI (Supplemental Figure 10, C and D). The similar domain structure further supported the notion that FMO2 protein might have a function similar to that of PDI in catalyzing disulfide bond formation and correct protein folding. We further performed structure-based sequence alignment of the thioredoxin-like domain (part of domain 1) from FMO2 and domains a/a′ from PDI. According to the Basic Local Alignment Search Tool (BLAST) results, the residue GVSG (from amino acids 11–14) in FMO2 was aligned with the CGHC active site motif position in PDI (Supplemental Figure 10E). This GVSG motif of FMO2 is conserved across different mammalian species. Analysis of hydrogen bond interactions revealed that the corresponding oxygen atoms on FAD form hydrogen bond interactions with G11, V12, and S13 sites on the GVSG motif (Supplemental Figure 10F). These interactions stabilize FAD within the FMO2 pocket, preventing FAD displacement during the reaction process.

To further understand the function of the GVSG motif, we generated FMO2-MUT (GVSG mutated to 4 alanines). The analysis of RMSD revealed that, compared with FMO2-WT, FMO2-MUT had notable fluctuations between 0 and 20 ns. After 360 ns, the structures of both FMO2-WT and FMO2-MUT stabilized, making them suitable for further protein comparison and protein tunnel analysis (Supplemental Figure 10G).

**Figure 8:**
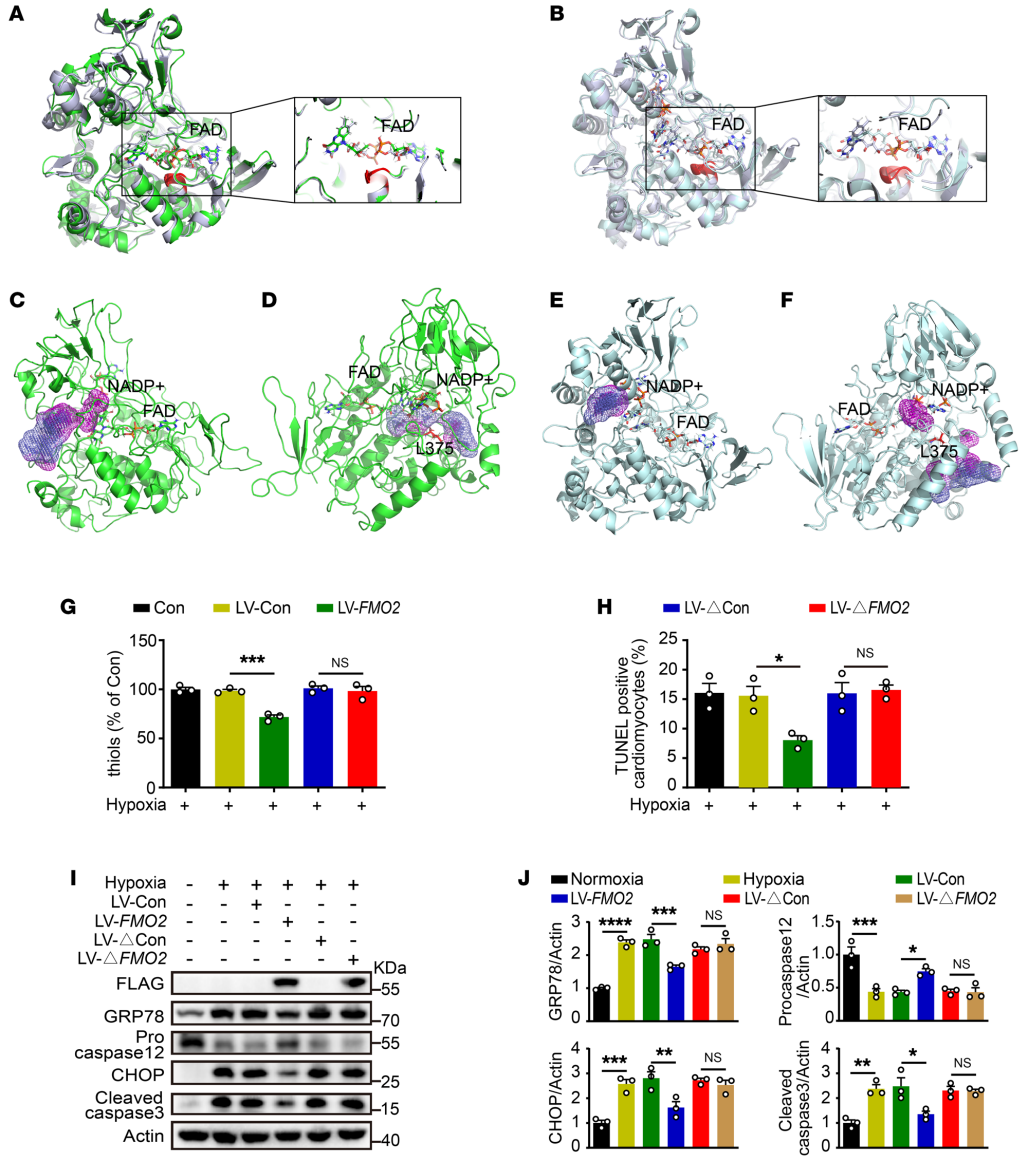
FMO2 depends on its GVSG motif to promote disulfide bond formation. (**A**) Structure of FMO2-WT (green) after 500 ns dynamic simulation, aligned with the original crystal structure of FMO2 (blue-white). (**B**) Structure of FMO2-MUT (pale cyan), which is the structure of FMO2 with the GVSG motif mutated to 4 alanines, after 500 ns dynamic simulation, aligned with the original crystal structure of FMO2 (blue-white). Red stick-and-ball represents oxygen atoms, blue stick-and-ball represents nitrogen atoms, and orange represents sulfur atoms. (**C**) In FMO2-WT, the substrate tunnel was constituted by the loop structure at positions 57–60 and the helix structure at positions 194–198. (**D**) In FMO2-WT, upon rotation as depicted in **C**, the tunnels are demonstrated with L375 colored in red. (**E**) In FMO2-MUT, the tunnel in the same orientation as in **C** is illustrated. (**F**) In FMO2-MUT, after rotation as shown, the tunnels are presented where L375, colored in red, obstructs the tunnel routes. (**G**) Contents of reduced thiols in hypoxia-exposed NRCMs with LV-*FMO2* or LV-Δ*FMO2*. Data are from the differently treated groups normalized to the hypoxia control. (**H**) Quantitative analysis of TUNEL staining of hypoxia-exposed NRCMs with LV-*FMO2* or LV-Δ*FMO2*. (**I** and **J**) Western blots (**I**) and analysis (**J**) of protein expression of GRP78 and ER stress–induced apoptotic proteins in hypoxia-exposed NRCMs with LV-*FMO2* or LV-Δ*FMO2*. The graphs summarize data from 3 independent experiments. Data are presented as the mean ± SEM, and significance was evaluated by 1-way ANOVA with Tukey’s test among 3 or more groups. *P < 0.05, **P < 0.01, ***P < 0.001, and ****P < 0.0001. ΔFMO2, GVSG-mutant FMO2.

After conducting dynamic simulations for 500 ns, we obtained stable structures of FMO2-WT and FMO2-MUT. We then compared each of these structures with the original crystal structure of FMO2. As shown in Figure 8A, FAD remained stably anchored in the binding pocket of FMO2-WT, with its relative relationship to the protein structure of FMO2-WT essentially unchanged. In contrast, as depicted in Figure 8B, although the horizontal position of FAD in FMO2-MUT did not appear to change markedly, it had undergone an axial displacement. This phenomenon may have been caused by losing the hydrogen bonds between FAD and the GVSG motif. Next, we observed that oxidative-reductive reactions involving NADP(H) and FAD occurred within the core reaction area of FMO2, with the oxidation of substrate molecules. Structural analysis revealed that the substrate molecules could access the core reaction area via 2 main tunnels, where they subsequently underwent oxidation due to FAD. One of these tunnels was situated between the loop structure at positions 57–60 and the helix structure at positions 194–198 (Figure 8, C and E), while the other tunnel was similar to that reported by Nicoll et al. (34), with the conserved homologous site L375 acting as a potential gatekeeper amino acid for this tunnel (Figure 8, D and F). On the basis of the stable structures of FMO2-WT and FMO2-MUT derived from dynamic simulations, we further analyzed the changes in these 2 tunnels. We found that FMO2-WT sustained the openness of both tunnels. FAD, not having undergone axial displacement, was situated near the tunnel opening formed by the loop structure at positions 57–60 and the helix structure at positions 194–198 (Figure 8C). The L375 site did not affect the patency of the tunnel (Figure 8D). In contrast, FMO2-MUT blocked the openness of both tunnels. FAD, because of the axial displacement, moved away from the tunnel opening formed by the loop structure at positions 57–60 and the helix structure at positions 194–198 (Figure 8E), and L375 began to act as a gatekeeper amino acid, thereby blocking the tunnel (Figure 8F).

Given the data collected, mutations in the GVSG motif of FMO2 could potentially affect the substrate oxidation of the enzyme via 2 plausible mechanisms: (a) the axial displacement of FAD could, on one hand, inhibit the reduction of FAD facilitated by NADPH and, on the other hand, lead to the removal of FAD from the tunnel entrance, ultimately hindering the oxidation of substrate molecules; and (b) the narrowing of the tunnel entrance between the loop structure at positions 57–60 and the helical structure at positions 194–198, along with the enhanced gatekeeping function of Leu375, restrict the access of substrate molecules to the core reaction region of FMO2. Together, we propose that GVSG in FMO2 serves as a crucial motif for regulating its enzymatic activity.

Moreover, in order to validate the role of GVSG in FMO2-mediated cytoprotection, we constructed the GVSG-mutant *FMO2*-overexpressing lentivirus (LV-Δ*FMO2*, GVSG mutated to 4 alanines). The expression of GVSG-mutant FMO2 (ΔFMO2) via the lentivirus had no catalytic activity with regard to disulfide bond formation (Figure 8G). TUNEL staining showed that ΔFMO2 overexpression failed to reduce hypoxia-induced apoptosis in CMs (Figure 8H and Supplemental Figure 11). In addition, ΔFMO2 had no effect on the suppression of GRP78 or CHOP, nor did it affect the cleavage of caspase 12 or caspase 3 (Figure 8, I and J). Hence, the role of FMO2 in disulfide bond synthesis and subsequent ER stress modulation is dependent on its GVSG motif.

### The therapeutic potential of FMO2 in infarcted hearts.

Having demonstrated the critical role of FMO2 in cardiac protection in response to cardiac injury, we next investigated whether FMO2 could serve as a potential therapeutic target for treating infarcted hearts. After MI surgery, rats were injected with either AAV9-*FMO2* or AAV9-Con. We demonstrated that AAV9-*FMO2* treatment effectively improved cardiac function at 4 and 6 weeks after MI (Supplemental Figure 12, A–D), decreased scar size (Supplemental Figure 12, E and F), and reduced ER stress (Supplemental Figure 12, G and H). These data suggest that AAV9-mediated FMO2 delivery is an effective therapeutic strategy for MI injury.

Moreover, we examined the role of FMO2 in a rat ischemia/reperfusion (I/R) model. AAV9-mediated, CM-specific FMO2 knockdown exacerbated CM apoptosis and ER stress 4 hours after reperfusion (Supplemental Figure 13, A–D). Twenty-eight days after I/R, rats treated with AAV9-sh*FMO2* showed reduced cardiac function (Supplemental Figure 13, E–H) and increased scar size (Supplemental Figure 13, I and J). Conversely, CM-specific overexpression of FMO2 mitigated I/R-induced CM apoptosis and ER stress (Supplemental Figure 14, A–D), leading to improved cardiac function and cardiac remodeling (Supplemental Figure 14, E–J). It is important to note that *AAV9-ΔFMO2* (GVSG-mutant) rats did not exhibit alleviation of ER stress (Supplemental Figure 14, A–D) or improved cardiac function and remodeling, demonstrating that the GVSG-dependent disulfide bond catalytic activity of FMO2 in CMs is the key mechanism for improving ER stress and cardiac function.

Next, we investigated the translational relevance of FMO2-mediated protective effects in hiPSC-CMs. Using hiPSC-CMs subjected to hypoxia, we found that overexpression of FMO2 suppressed GRP78 expression and the ER stress–induced apoptotic response (Supplemental Figure 15, A and B), whereas FMO2 knockdown decreased FMO2 expression (Supplemental Figure 15, C and D) and had the opposite effects (Supplemental Figure 15, E and F). These results suggest that FMO2 confers a protective effect against ER stress and ER stress–induced apoptosis in human CMs, highlighting the clinical relevance for MI treatment.

We further explored the association between genetically predicted *FMO2* expression levels and the risk of HF. Mendelian randomization (MR) analysis is a commonly used method in epidemiology and genetics to study the causal effect of exposure on outcomes such as disease risk (35–37). We selected SNPs of the left ventricle from the Genotype-Tissue Expression (GTEx) Consortium (V8 release) as the exposure, genome-wide association study (GWAS) of patients with HF from the HERMES (Heart Failure Molecular Epidemiology for Therapeutic Targets) Consortium (47,309 patients and 930,014 controls) (38) as the outcome. SNPs from the GTEx consortium were extracted according to 2 criteria to ensure strong correlation and independence: the genome-wide level of statistical significance (*P* < 5 × 10^–8^) and linkage disequilibrium (LD) with *r^2^* < 0.001 and a clump window >10,000 kb. One SNP (rs78893152), strongly associated with *FMO2*, was identified and selected as the genetic variant for MR analysis. The results are listed in Supplemental Table 1. Higher genetically predicted *FMO2* expression levels in the left ventricle were causally associated with a reduced risk of HF (OR, 0.914; 95% CI, 0.841–0.993; *P* =0.034).

## Discussion

ER dysfunction plays an important role in ischemic injury, which is responsible for CM death. Proteins in the ER require disulfide bond formation, finally to be the mature state (7, 10). However, pathophysiological events occurring in MI, such as ischemia and nutrient deprivation, impair ER function and subsequently cause the accumulation of misfolded or unfolded proteins, leading to ER stress (19, 39). As ER stress is prolonged, the “adaptive UPR” becomes a “terminal UPR” that leads to CM apoptosis and eventually HF (19, 40). Therefore, specific regulation of ER stress is increasingly recognized as a promising therapeutic target for MI treatment (19, 39, 41). In this study, we found that FMO2 catalyzed disulfide bond formation, thereby inhibiting the UPR pathway to inhibit CM apoptosis during MI. CM-specific overexpression of FMO2 ameliorated MI injury, whereas FMO2 knockdown deteriorated cardiac dysfunction and remodeling. In CMs, FMO2 was identified as an ER chaperone that catalyzes disulfide bond synthesis in unfolded or misfolded proteins in a GVSG motif–dependent manner. The translational relevance of this finding was further supported by evidence showing the protective effects of human FMO2 against ER stress and ER stress–induced apoptosis in hiPSC-CMs. Taken together, this study provides mechanistic insights into the role of FMO2 in oxidative protein folding and highlights the critical function of this enzyme in ischemic heart diseases.

Since their discovery, FMOs have been reported to be associated with multiple human diseases. FMO3 mediates the metabolism of trimethylamine (TMA), causing fish odor syndrome (42). FMO1 levels decrease in the spinal cord of patients with amyotrophic lateral sclerosis (43), but increase in the myocardial tissue of patients with atrial fibrillation (44). Nevertheless, there are few studies on other FMO family orthologs, especially FMO2. In our recent study, we reported that FMO2 expression is induced in CMs after MI but is decreased in fibroblasts (17). In this study, we confirmed these findings in both rat models and heart tissue samples from patients with MI. FMO2 expression in CMs markedly increased after hypoxia and was associated with elevated HIF1 levels. This was consistent with previous studies showing that HIF1 activation upregulates FMO2 expression (45, 46). Interestingly, we also found that, despite the elevated expression of HIF, FMO2 expression was decreased in fibroblasts. We hypothesized that this was primarily due to the involvement of TGF-β in downregulating FMO2 expression in fibroblasts, as demonstrated in our previous research (17). In CMs, the increase in FMO2 expression induced by HIF1 mediated the synthesis of disulfide bonds in unfolded or misfolded proteins through its enzymatic activity.

Moreover, we revealed that FMO2 depended on its GVSG motif for disulfide bond synthesis. Our structural analysis, along with in vitro and in vivo experiments, substantiated the pivotal role of GVSG in this process. FMO2 with GVSG mutation failed to catalyze disulfide bond formation, resulting in the loss of the ability of FMO2 to alleviate ER stress and improve cardiac function following MI. Our data highlight the important role of the GVSG motif in FMO2-mediated disulfide bond formation for the targeted molecules, however, our findings do not exclude the possibility that some other important structures could also be involved in FMO2-mediated biological functions. As a chaperone, FMO2 ensures the proper folding of disulfide bond–containing proteins, thereby reducing the ER stress pathway and ultimately halting the progression of MI and HF. In summary, owing to the importance of FMO2 in the regulation of protein folding, we believe FMO2-based molecular therapy is a promising approach to treating a wide range of ER stress–related diseases, particularly MI.

This study revealed that AAV9-mediated FMO2 delivery could effectively treat infarcted rat hearts, improving cardiac dysfunction and adverse remodeling, underscoring its clinical relevance for MI treatment. We further validated the protective effect of FMO2 in hiPSC-CMs, a more translationally relevant model. High expression of FMO2 reduced ER stress and the ER stress–induced apoptotic response, whereas FMO2 knockdown had the opposite effect. On the basis of clinical data from a total of 977,323 individuals (47,309 patients with HF and 930,014 controls), we investigated the relationship between *FMO2* expression and HF. MR analysis showed that higher genetically predicted *FMO2* expression levels were causally associated with a reduced risk of HF. These findings suggest that FMO2 could serve as a therapeutic target in the treatment of patients with MI. Therefore, targeted delivery of FMO2 to the heart using extracellular vesicles or alternative carriers such as AAV9 could be an effective therapeutic strategy for MI. Designing a small peptide that targets CMs and fibroblasts to increase FMO2 expression within these cells could also be an important approach for achieving clinical translation of FMO2 and will be the focus of our future research. Additionally, screening for clinical drugs that increase FMO2 expression or agonists that enhance FMO2 enzymatic activity represents another promising strategy for treating patients with MI.

There are several limitations to the present study. First, although FMO2 inhibits ER stress and promotes cell survival in vitro, the effects of GVSG-mutated FMO2 on ER stress and organ function in animal models still need to be further investigated. Second, we used the CM-specific *FMO2*-knockdown AAV9 vector and global *FMO2*–KO rats to assess the effect of FMO2 deficiency on cardiac function. A CM-specific *FMO2*-KO rat model should be used, but such a model is not currently available.

In conclusion, we demonstrate that FMO2 ameliorated CM apoptosis and cardiac dysfunction during MI through a mechanism that involved suppression of the UPR pathway. This function relied on the enzymatic activity of FMO2, which catalyzed disulfide bond formation via its GVSG motif. We uncovered FMO2-involved regulatory mechanisms in oxidative protein folding and believe FMO2 has important therapeutic potential for the treatment of ischemic heart diseases.

## Methods

### Sex as a biological variable.

Our study examined male rats because male animals exhibited less variability in phenotype.

### Animals.

Male adult SD rats (3–8 weeks old) and neonatal SD rats (1–3 days old) were purchased from Shanghai Slac Laboratory Animal Technology Corporation. The *FMO2*-KO rat with a specific region deleted between 2 small guide RNAs was constructed by Biocytogen. One small guide RNA was located before exon 1 or exon 2, and the other was located after exon 9 or the 3′-UTR. Western blots and genotyping were performed to confirm this strategy. All animals were fed a standard laboratory diet under controlled conditions and were maintained on a 12-hour light/12-hour dark cycle.

### MI and I/R models.

MI was surgically induced as described previously (47). Male SD rats were anesthetized by intraperitoneal injection of pentobarbital sodium (60 mg/kg) and ventilated via tracheal intubation. MI was induced by ligation of the left anterior descending coronary artery using a 6-0 nylon suture (Huawei Medical Instruments), confirmed by a pale area below the suture. The heart was re-placed immediately into the thoracic cavity, and the chest was closed. Rats in the sham treatment group were subjected to the same procedures for MI surgery except for the ligation. Body temperature was maintained at 37°C with a heating pad and monitored with a thermometer. For the I/R surgery, we encircled the left anterior descending coronary artery with a 6-0 nylon suture and ligated the artery through a vinyl tube. After 45 minutes of ischemia, we released the ligation and reperfused the heart. The sham treatment group underwent the same procedure without ligation.

### Culturing of adult and neonatal rat CMs.

Adult rat CMs (ARCMs) were isolated from 2-month-old SD rat hearts as previously described (48). The descending aorta was cut and the right ventricle was injected with 20 mL EDTA buffer (MilliporeSigma). Next, the ascending aorta was clamped and the heart was excised and processed by sequential injection of EDTA buffer, perfusion buffer as well, as collagenase buffer (Gibco, Thermo Fisher Scientific) into the left ventricle. The respective chambers were separated and pulled into 1 mm^3^ pieces using forceps. After the addition of stop buffer, the cell suspension was passed through a 100 μm filter, followed by gravitational settling. The cell pellet was enriched for CMs. NRCMs from 1- to 3-day-old SD rat hearts were isolated and cultured by enzymatic digestion, as described previously (49). Briefly, hearts were minced, digested with 0.05% w/v Trypsin/EDTA (Genomcellbio Biotechnology), and 0.05% w/v type II collagenase (Gibco, Thermo Fisher Scientific). The digestion was centrifuged and plated for 90 minutes at 37°C with 5% CO_2_. The cells were cultured with 10% FBS-supplemented high-glucose DMEM (Corning) containing 5-bromo-2-deoxyuridine (MilliporeSigma) on plates coated with 0.5% gelatin (MilliporeSigma).

### Hypoxia culture conditions.

Cells were maintained with glucose- and serum-free medium in a hypoxia chamber (0.5% O_2_, 5% CO_2_, BioSpherix) for 24 or 48 hours. For H/R, cells were exposed to hypoxia and then replaced with complete culture medium under normoxic conditions.

### Human heart samples and ethics statement.

Left ventricles were obtained from patients with MI undergoing heart transplantation, and the control samples were obtained from patients who died from brainstem bleeding.

### AAV9 construction and injection.

To specifically knock down expression of FMO2 in CMs, a cardiac-specific troponin T promoter (cTnT) was used to generate AAV9-cTnT-*FMO2* shRNA (AAV9-sh*FMO2*) and AAV9-cTnT-shCon (AAV9-shCon). An shRNA targeting *FMO2* mRNA or a nonspecific shRNA was cloned into CAG-miR30-GFP plasmids. We then used a previously described method (50) to make an AAV that simultaneously expressed *FMO2* shRNA. To specifically induce overexpression of FMO2 in CMs, we constructed AAV9-cTnT-*FMO2*-3FLAG (AAV9-*FMO2*), AAV9-cTnT-GVSG mutant *FMO2*-3FLAG (AAV9-Δ*FMO2*), and AAV9-cTnT-luciferase 2 (AAV9-Con). The cDNA fragments encoding rat *FMO2*, Δ*FMO2*, and luciferase 2 were separately cloned into the inverted terminal repeats–containing (ITR-containing) AAV plasmid (Penn Vector Core) harboring the cTnT promoter to generate pAAV.cTnT:*FMO2*-3FLAG, pAAV.cTnT:Δ*FMO2*-3FLAG, and pAAV.cTnT:luciferase 2. The AAV was packaged into an AAV9 capsid using AAV9:Rep-Cap and pAd deltaF6 (Penn Vector Core) and purified and concentrated by gradient centrifugation (51). Three-week-old male rats received a single injection of AAV9 (4 × 10^12^ viral genome particles per rat) via the tail vein. Four weeks later, the rats were subjected to MI surgery.

### Echocardiography.

Echocardiography was performed on days 0, 3, and 28 after MI surgery. Two-dimensional and M-mode echocardiographic images were obtained and analyzed with the Vevo 2100 system. The left ventricular internal diameter at end-diastole (LVIDd) and left ventricular internal diameter at end-systole (LVIDs) were measured for at least 3 separate cardiac cycles per rat. Fractional shortening (FS) was calculated as follows: (LVIDd – LVIDs)/LVIDd × 100. The EF was calculated as follows: (LVEDV – LVESV)/LVEDV × 100, where LVEDV is the left ventricular end-diastolic volume and ESV is the end-systolic volume.

### Western blot analysis.

Cells and tissues were lysed in RIPA buffer (Beyotime Biotechnology) containing a protease inhibitor cocktail and a phosphatase inhibitor cocktail (Roche). Then, the lysates were collected by centrifugation at 13,000*g* for 30 minutes at 4°C, and the protein concentration was determined by bicinchoninic acid (BCA) assay (Pierce, Thermo Fisher Scientific). Equal amounts of proteins were separated by 12% SDS–PAGE and electrophoretically transferred to PVDF membranes. The membranes were blocked with 5% BSA for 1 hour at room temperature and incubated overnight with the relevant primary antibodies against FMO2 (1:500, NBP1-85952, Novus Biologicals), FMO1 (1:1,000, ab97720, Abcam), FMO3 (1:1,000, ab126711, Abcam), FMO4 (1:1,000, ab191141, Abcam), FMO5 (1:1,000, ab189516, Abcam), cardiac troponin I (1:500, ab47003, Abcam), FLAG-tag (1:1,000, ab49763, Abcam), cleaved caspase 3 (1:500, 9661, Cell Signaling Technology), GRP78 (1:1,000, ab21685, Abcam), caspase 12 (1:200, C7611, MilliporeSigma), CHOP (1:200, sc-7351, Santa Cruz Biotechnology), p-PERK (1:500, 3179, Cell Signaling Technology), PERK (1:500, 3192, Cell Signaling Technology), p-eIF2α (1:1,000, 9721, Cell Signaling Technology), eIF2α (1:1,000, 5324, Cell Signaling Technology), ATF6 (1:500, 24169, Proteintech), sXBP1 (1:1,000, 619502, BioLegend), PDI (1:1,000, 3501, Cell Signaling Technology), HIF1 (1:1,000, ER1802-41, Huabio), and actin (1:10,000, KC-5A08, Kangchen). Subsequently, membranes were reacted with HRP-conjugated secondary antibodies for 1 hour at room temperature and detected using an ECL kit (MilliporeSigma).

### Quantitative reverse transcription PCR analysis.

Total RNA was extracted with TRIzol (Invitrogen, Life Technologies, Thermo Fisher Scientific) according to the manufacturer’s protocol. For reverse transcription, RNA samples were mixed with oligo dT and MMLV reverse transcriptase (TAKARA) in a 20 μL reaction. Quantitative reverse transcription PCR (qRT-PCR) was performed using SYBR Premix Ex Taq (Takara) under conditions of 40 cycles at 95°C for 30 seconds and 60°C for 30 seconds. mRNA expression was normalized to rat *β-actin*. The following mRNA primers were used: rat *FMO2*, forward, 5**′**-TGCGTGCATAGGTCTCATCC-3′, reverse, 5′-CAAGGCGAGTTCATCCAGGT-3′; rat *β-actin*, forward, 5′-ATGACGATATCGCTGCGCTC-3′, reverse, 5′-CAGTTGGTGACAATGCCGTG-3′; rat *PDI*, forward, 5′-TGCGGCTTATTACCCTGGAG-3′, reverse, 5′-CATCAGGTGGGGCTTGATCTT-3′.

### Immunofluorescence and Masson’s trichrome staining.

Hearts were excised from anesthetized rats, dehydrated in 30% sucrose solution (Sangon Biotech), embedded with Tissue-Tek OCT compound (Sakura), and cut into 7 μm sections from the apex to the mid-papillary (800 μm intervals). The heart tissue sections were fixed in 4% paraformaldehyde, permeabilized with 0.5% Triton X-100, and blocked in 5% BSA. Wheat germ agglutinin (WGA) (Thermo Fisher Scientific) and anti–troponin I antibody (ab47003, Abcam) were used to mark the CMs in the infarcted area. A goat anti-rabbit secondary antibody (ab96883, Abcam) was used for visualization under fluorescence microscopy (Leica). For Masson’s trichrome staining, tissue sections were stained using a commercially available kit (Solaribio). The scar area was calculated by the sum of the endocardial and epicardial lengths of the scar zone divided by the sum of the endocardial and epicardial lengths of the left ventricle using Imaging Pro Plus software.

### TUNEL assay.

For the detection and quantification of apoptosis, TUNEL assays were performed using a TUNEL kit (Roche). Tissue or cell samples were treated in accordance with the manufacturer’s protocol and costained with rabbit anti–troponin I (1:200) and DAPI (1:5,000). Apoptosis was determined according to the percentage of apoptotic CM nuclei among total CM nuclei in 6–9 randomly selected fields per heart or at least 5 fields per well.

### Lentivirus construction and transfection.

Lentiviral plasmids encoding the shRNA targeting rat *FMO2* mRNA (LV-sh*FMO2*) and its nonspecific shRNA (LV-shCon) were generated by Genechem. To upregulate FMO2 expression in CMs, we constructed the full rat *FMO2*-3FLAG (LV-*FMO2*), rat mutated *FMO2*-3FLAG (LV-mutated *FMO2*, 9-14/40-41/60-62/191-198aa deletion), or rat GVSG-mutant *FMO2*-3FLAG, in which GVSG was replaced by 4 alanines (LV-Δ*FMO2*) expressing lentivirus, while an empty lentiviral plasmid served as the control (Genechem). To induce overexpression of FMO2 in human CMs, we constructed the full human *FMO2*-3FLAG–expressing (LV-h*FMO2*–expressing) lentivirus, and an empty lentiviral plasmid served as the control (Genechem). To induce overexpression of CHOP, we constructed the *CHOP*-3FLAG–expressing (LV-*CHOP*-expressing) lentivirus, and an empty lentiviral plasmid served as the control (Genechem). Cells were transfected with lentivirus for 12 hours and the medium was replaced. After 72 hours of infection, the efficiency of the infection was assessed by Western blotting.

### siRNA synthesis and transfection.

siRNAs targeting rat *PDI* and human *FMO2* were designed and synthesized by GenePharma. The targeting siRNA and the scrambled siRNA control were transfected into cells at a 50 nM final concentration using Lipofectamine RNAiMAX Reagent (Thermo Fisher Scientific).

### RNA-Seq and data analysis.

RNA-Seq was performed by Novogene. Briefly, mRNA was purified from total RNA using poly-T oligo-attached magnetic beads. Fragmentation was carried out using divalent cations under elevated temperature in First Strand Synthesis Reaction Buffer (5×). With the help of the NEBNext Ultra RNA Library Prep Kit from Illumina, sequencing libraries were generated. The clustering of the index-coded samples was performed on a cBot Cluster Generation System using TruSeq PE Cluster Kit v3-cBot-HS (Illumina). After cluster generation, the library preparations were sequenced on an Illumina NovaSeq platform, and 150 bp paired-end reads were generated. For data analysis, raw data (raw reads) in fastq format were first processed through in-house Perl scripts. Clean data (clean reads) were obtained by removing reads containing adapter, reads containing ploy-N, as well as low-quality reads from raw data. At the same time, Q20, Q30, and GC content of the clean data was calculated. Reference genome and gene model annotation files are available at the Ensembl genome website (https://www.ensembl.org/). The index of the reference genome was built, and paired-end clean reads were aligned to the reference genome by Hisat2 version 2.0.5. FeatureCounts version 1.5.0-p3 was used to count the read numbers mapped to each gene, and then the fragments per kilobase of transcript per million mapped reads (FPKM) was calculated on the basis of the length of the gene and read count mapped to this gene. Analysis of differential expression between 2 groups was performed using the DESeq2 R package (1.20.0). *P* values were adjusted using the Benjamini and Hochberg methods for controlling the FDR. Genes with an adjusted *P* value of less than 0.05 found by DESeq2 were designated as differentially expressed. GO and KEGG enrichment analysis of differentially expressed genes was implemented by the clusterProfiler R package, in which the gene length bias was corrected. GO terms and KEGG pathways with a corrected *P* value of less than 0.05 were considered significantly enriched by differentially expressed genes.

### Transmission electron microscopy.

As described before (49), samples of heart tissue or NRCMs were fixed in 2.5% glutaraldehyde, postfixed in osmium tetroxide, stained with uranyl acetate, dehydrated by an ethanol gradient, and embedded in epoxy resin. Finally, the samples were cut into ultrathin sections and captured randomly for images using a H-7500 transmission electron microscope (Hitachi).

### DTT-induced reductive stress model.

As described previously (25), cells were subjected to a brief 4-hour pulse of 5 mM DTT (MilliporeSigma) and then replaced with fresh media for 2 hours. Cell viability was detected using a Cell Counting Kit-8 (CCK-8, C0037, Beyotime Biotechnology), and the absorbance was measured at 450 nm using a spectrophotometer (Molecular Devices) in accordance with the manufacturer’s protocol.

### Determination of the thiol group.

To measure the thiol content in cells, a total thiol group detection kit (BestBio) was used. After grinding, the cell supernatant was added to Ellman’s reagent (DTNB) and incubated at 25°C for 10 minutes, following the manufacturer’s protocol. The absorbance at 412 nm was measured using a spectrophotometer (Molecular Devices). A control was conducted by replacing the sample with 500 μM GSH (MilliporeSigma).

### Thiol-disulfide exchange reactions.

For reaction with the substrate GSH, the mixtures contained 0.1 M tricine (MilliporeSigma), pH 8.4, 1.0 mM EDTA, 80 μM GSH, 0.1 mM NADPH (MilliporeSigma), and 0.3 mM DTNB (MilliporeSigma), with or without addition of 3 μM purified FMO2 (Sino Biological), for a final volume of 200 μL at 37°C. GSH and GSSG levels were measured using the GSH/GSSG Assay Kit (MilliporeSigma, MAK440).

As described previously (27, 28), oxidation of the substrate 5 μM peptide NRCSQGSCWN (Chinese Peptide) was reacted with McIlvaine buffer (0.2 M disodium hydrogen phosphate/0.1 M citric acid, pH 3.0–7.5), 0.5 mM GSSG (MilliporeSigma), 2 mM GSH, and 0.1 mM NADPH, with or without addition of 1 μM FMO2. The fluorescence intensity (excitation 280 nm, emission 350 nm) was monitored over an appropriate period of time.

### Determination of the redox state of PDI.

The PDI redox state was assessed by its electrophoretic migration shift after alkylation of the thiol groups with AMS (Invitrogen, Thermo Fisher Scientific). Cells were incubated for 20 minutes with or without DTT (10 mM), washed in ice-cold PBS containing 20 mM *N*-ethylmaleimide (NEM) (MilliporeSigma), lysed in RIPA buffer containing 12 mM TCEP (Sangon Biotech), and treated with 25 mM AMS for 60 minutes at room temperature in the dark. Samples were boiled for 4 minutes and used for nonreducing SDS-PAGE and further Western blotting.

### Structural analysis.

The structure of FMO2 in complex with FAD and NADP+ was obtained from the Protein Data Bank (PDB ID: 6SF0). Missing residues were modeled by the ModLoop program, and the protonation state of each residue was determined using PDB2PQR software. The protein parameters were obtained from the ff14SB force field in the AMBER software program, while FAD and NADP+ were optimized for structure at the AM1 level, and the charges of each atom were assigned by bond charge correction (BCC) using the antechamber module, whose parameter files were generated on the basis of the general amber force field (GAFF). Subsequently, the complex was solvated within a periodic TIP3P water box with a buffer distance of 12 Å between the complex and the box edges. To reflect the physiological environment, a 150 mM NaCl solution was used, and an appropriate amount of Cl^–^ ions were added to maintain a neutral system. Ionic parameters were adopted from the work of Joung and Cheatham (52). The energy minimization of the system was carried out by the most rapid descent algorithm mixed with the conjugate gradient algorithm. Then, the system was gradually heated from 0 K to 300 K under a restraint force of 10 kcal/(mol × Å2) in the number, volume, and temperature (NVT) ensemble. Finally, the system was switched to the number, pressure, and temperature (NPT) ensemble for a 500 ns unconstrained molecular dynamics simulation with a time step of 2 fs. The temperature was regulated by Langevin dynamics with a collision frequency of 1 ps^–1^, and the SHAKE algorithm was applied to constrain bonds involving hydrogen atoms. PyMOL software was used for protein structure visualization. The diagrams of sequence comparison and analysis were generated with ESPript 3.0 (53).

### Generation of CMs from hiPSCs.

The method to generate CMs was used as previously described (18). Briefly, hiPSCs (HELP Stem Cell Innovations) were routinely cultured in Matrigel-coated plates in mTeSR1 medium (STEMCELL Technologies). The cells at 80%–90% confluence were dissociated with Accutase (STEMCELL Technologies) and plated onto Matrigel-coated 12-well plates in mTeSR1 plus 5 μM Y27632 (Tocris) at a density of 1 × 10^4^ cells/cm^2^. Old medium was replaced with fresh mTeSR1 every day. After 3 days, differentiation was induced by culturing hiPSCs with 6 μM CHIR99021 (Selleckchem) plus RPMI/B-27 without insulin. Forty-eight hours later, the cells were maintained in RPMI/B-27 without insulin. Then, the cells were cultured in 5 μM IWP2 (Biorbyt) plus RPMI/B-27 without insulin for 48 hours. Spontaneous contraction usually occurred approximately 12 days after differentiation was initiated, and hiPSC-CMs were purified and enriched in glucose-depleted medium with abundant lactate.

### Characterization of hiPSC-CMs.

hiPSC-CMs were characterized according to expression of the markers troponin I and α-sarcomeric actinin (α-actinin). Briefly, the cells were fixed in 4% paraformaldehyde, permeabilized with 0.5 % Triton X-100, and then blocked in 5% BSA. Next, hiPSC-CMs were incubated with rabbit anti–troponin I antibody (1:500, ab47003, Abcam) and mouse anti–α-actinin (1:200, ab9465, Abcam) primary antibodies; then, corresponding fluorescently conjugated secondary antibodies were used for visualization under fluorescence microscopy. Nuclei were stained with DAPI. For flow cytometric analysis, hiPSC-CMs were fixed and permeabilized using the True-Nuclear Transcription Factor Buffer Set (BioLegend) and then labeled with phycoerythrin (PE) mouse anti–troponin T antibody (1:100, 564767, BD Biosciences) for 15 minutes at 4°C. Cell fluorescence was assessed using a BD FACSCanto II Flow Cytometer (BD Biosciences).

### Statistics.

All statistical analyses were performed using GraphPad Prism 8.0 (GraphPad Software). For the in vitro study, all biological replicates using cultured cells correspond to independent experiments from distinct expansions and passage numbers, with technical replicates (the exact replicate number is indicated in the figure legends). The Shapiro-Wilk test (*P* < 0.05) was used to test the normality of all data obtained from the in vivo study. Comparisons between 2 groups were assessed by 2-tailed Student’s *t* test; comparisons among 3 or more groups were evaluated using a 1-way ANOVA with Tukey’s test; and comparisons among groups after multiple treatments were evaluated using a 2-way ANOVA with Tukey’s test. Data are presented as the mean ± SEM. A *P* value of less than 0.05 was considered statistically significant.

### Study approval.

Animal experiments were conducted in compliance with *The* *Care and Use of Laboratory Animals* published by the NIH (NIH publication no. 85-23, National Academies Press, revised 1996) and approved by the IACUC of Zhejiang University. Human studies were approved by the ethics committee of the Second Affiliated Hospital of Zhejiang University. All participants were duly informed, and written consent was obtained from the patients or their relatives.

### Data availability.

All data associated with this study are present in the manuscript or in the supplemental materials and are available in the Supporting Data Values file. RNA-Seq data have been deposited in the BioProject database (PRJNA1131740, https://www.ncbi.nlm.nih.gov/bioproject/?term=PRJNA1131740; PRJNA1131998, https://www.ncbi.nlm.nih.gov/bioproject/?term=PRJNA1131998).

## Author contributions

JW and X Hu conceived of and designed the study. QL, JH, and HD designed the experiments, analyzed data, and drafted the manuscript. YT, CX, YW, ZZ, X Hu, and CZ acquired in vitro and in vivo data. JN, CN, DX, and X He analyzed and interpreted the data. RW, WZ, JC, and X Hu reviewed and edited the manuscript. QL, JH, HD, and YT share first authorship, and the order in which they are listed was determined by workload.

## Supplementary Material

Supplemental data

Unedited blot and gel images

Supporting data values

## Version 1 10/31/2024

In-Press Preview

## Version 2 12/16/2024

Electronic publication
