# Supplementary material for: Flavin-containing monooxygenase 2 confers cardioprotection in ischemia models through its disulfide bond catalytic activity
Source: J Clin Invest. 2024 Oct 31;134(24):e177077. doi: 10.1172/JCI177077 (PMC11645147; doi:10.1172/JCI177077)
Supplement: Supplemental data [file jci-134-177077-s222.pdf]

Supplemental materials

¬Flavin-containing monooxygenase 2 confers cardioprotection in ischemia models through its disulfide-bond catalytic activity

Qingnian Liu, Jiniu Huang, Hao Ding, Yue Tao, Jinliang Nan, Changchen Xiao, Yingchao Wang, Rongrong Wu, Cheng Ni, Zhiwei Zhong, Wei Zhu, Jinghai Chen, Chenyun Zhang, Xiao He, Danyang Xiong, Xinyang Hu, Jian'an Wang

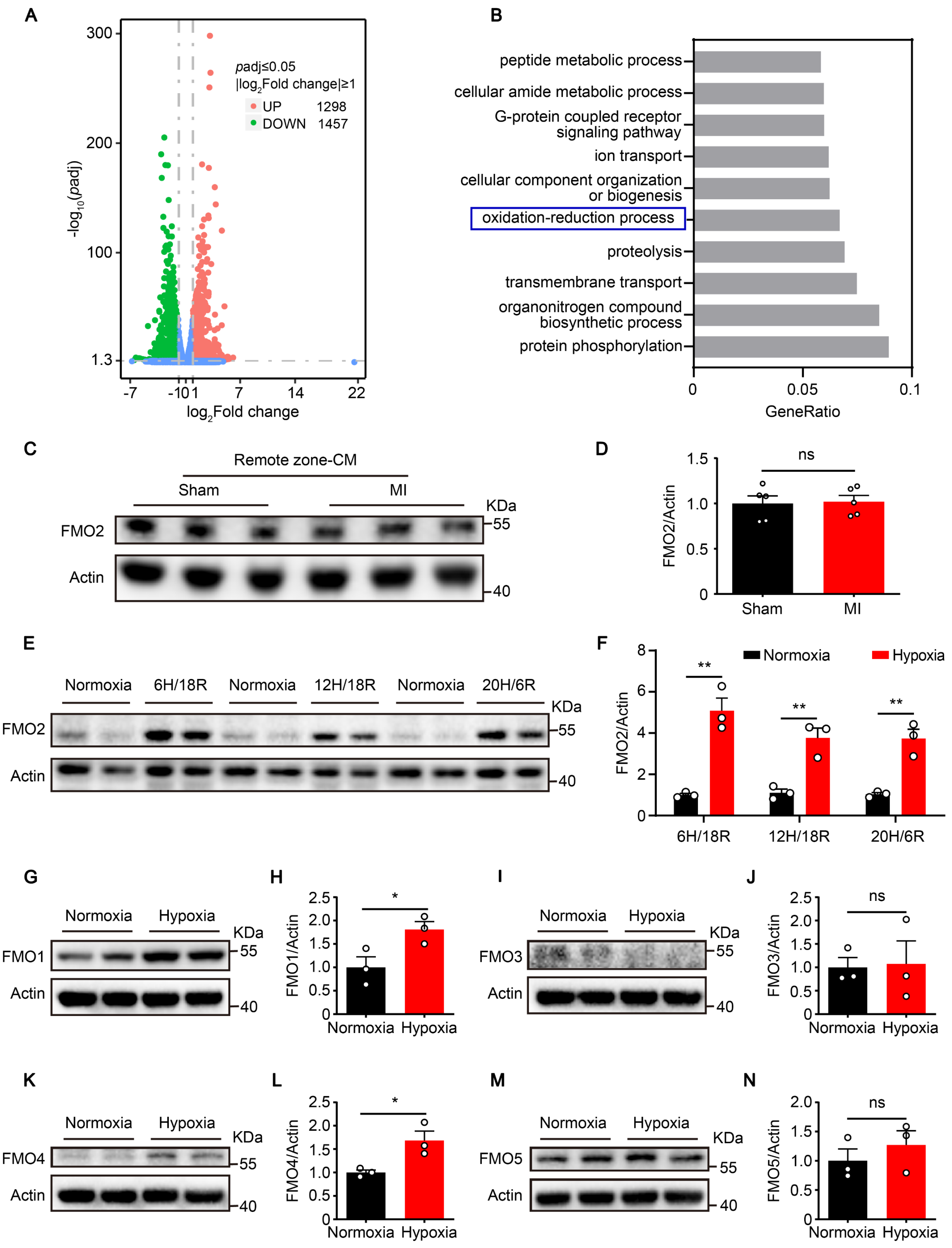

Supplemental Figure 1. Expression of FMOs in cardiomyocytes upon hypoxia. (A) Volcano map showed differentially expressed genes in hypoxia NRCMs compared with

normoxia NRCMs. (**B**) Pathway enrichment analysis of significantly altered genes identified between hypoxia and normoxia NRCMs. (**C** and **D)** Expression of FMO2 in CMs isolated from the remote zone of adult normal and infarcted rat hearts (n = 5 per group). (**E** and **F**) Expression of FMO2 in NRCMs under classical hypoxia/reoxygenation injuries. (**G-N**) Protein expression of FMO1, FMO3, FMO4 and FMO5 in NRCMs subjected to hypoxia for 24 hours. The graphs summarize data from 3 independent experiments. Quantified data are presented as means ± SEM, and significance was evaluated via t test. *p<0.05, **p<0.01, ns: not significant. CM indicates cardiomyocyte.

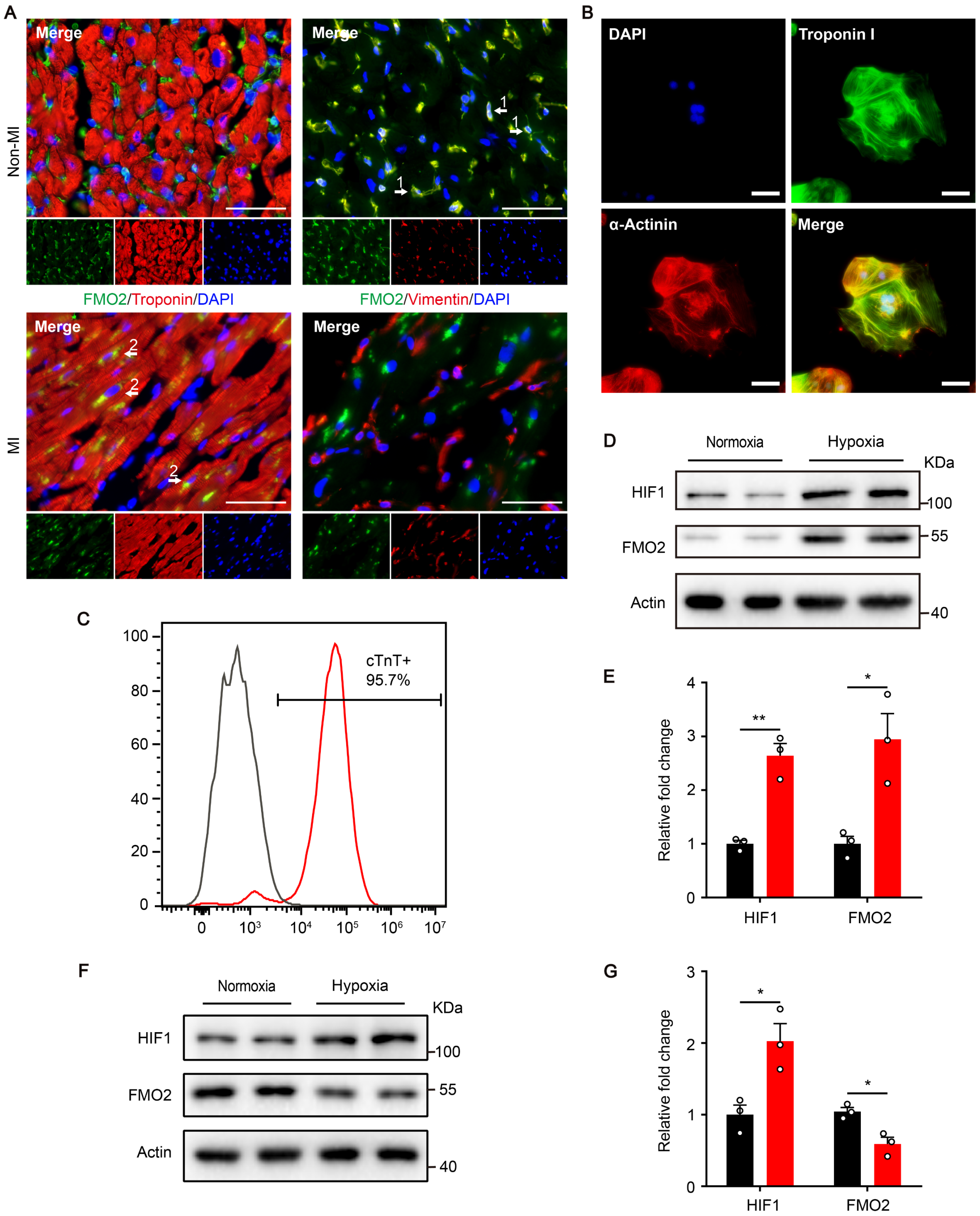

Supplemental Figure 2. Expression of FMO2 in different cardiac cells. (A) Immunofluorescence staining for FMO2 (green in respective image), Troponin I (cardiomyocyte marker), and Vimentin (fibroblast marker) in heart section from MI patients

and non-MI controls. Arrow 1 indicates FMO2-positive fibroblast, and arrow 2 indicates FMO2-positive cardiomyocyte. Bar = 50 μm. (**B**) hiPSC-CMs were characterized via immunofluorescent analyses of Troponin I and α-Actinin. Bar = 100 um. (**C**) The purity of hiPSC-CMs that expressed Troponin T was determined via flow cytometry (n = 3 independent experiments). (**D** and **E**) Protein expression of HIF1 and FMO2 in hiPSC-CMs subjected to hypoxia. (**F** and **G**) Protein expression of HIF1 and FMO2 in neonatal rat cardiac fibroblasts under hypoxia. The graphs summarize data from 3 independent experiments. Quantified data are presented as means ± SEM. Comparisons between two groups were assessed via the Student's t-test. *p<0.05, **p<0.01. α-Actinin indicates α-sarcomeric actinin. hiPSC-CMs indicates human-induced pluripotent stem cell–derived cardiomyocytes.

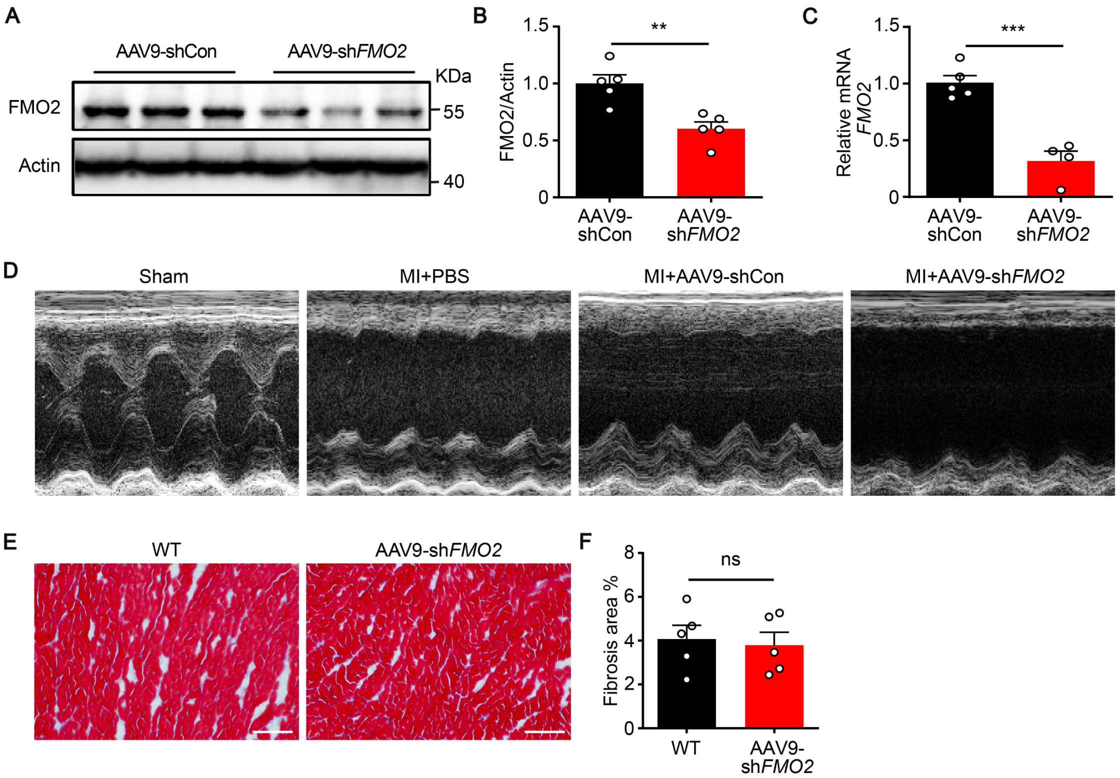

Supplemental Figure 3. Effect of AAV9-shFMO2 in rat hearts. (**A-C**) FMO2 protein and mRNA levels in CMs isolated from control and AAV9-shFMO2 rat hearts (n = 4-5 per group). (**D**) Representative photographs of M-mode echocardiography. (**E** and **F**) Masson staining showing different degrees of fibrosis in heart from WT and AAV9-shFMO2 rats. Bar = 100 μm. The summary data of fibrosis are shown in (right, n = 5 per group). Quantified data are presented as means ± SEM. Comparisons between two groups were assessed via the Student's t-test. ***p*<0.01, ****p*<0.001, ns: not significant.

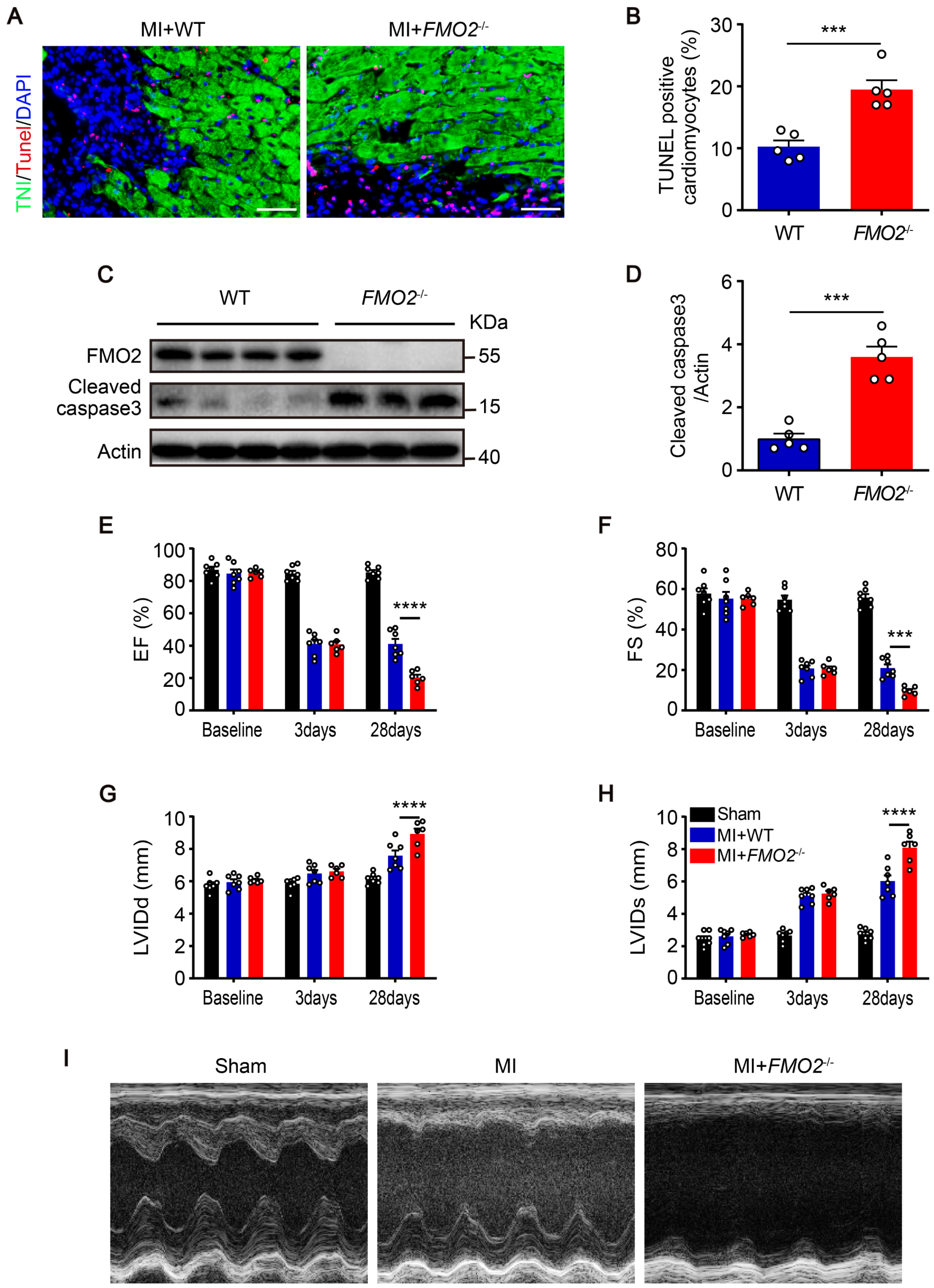

Supplemental Figure 4. Knockout of FMO2 impairs cardiac function accompanied by increased cardiomyocyte apoptotic level after MI. (**A** and **B**) Analysis of TUNEL-Troponin I positive cardiomyocytes in the border zone of WT and *FMO2*$^{-/-}$ infarcted rat hearts (n = 5 per

group). Bar = 50 μm. (**C** and **D**) Protein expression of cleaved caspase 3 in each group of rats as described above (n = 5 per group). (**E-H**) Quantitative analysis of echocardiography in each group of rats (n = 6-7 per group). (**I**) Representative photographs of M-mode echocardiography. Quantified data are presented as means ± SEM. Comparisons between two groups were assessed via the Student's t-test, comparisons among groups after multiple treatments were evaluated via two-way ANOVA with Tukey test. ***p<0.001, ****p<0.0001.

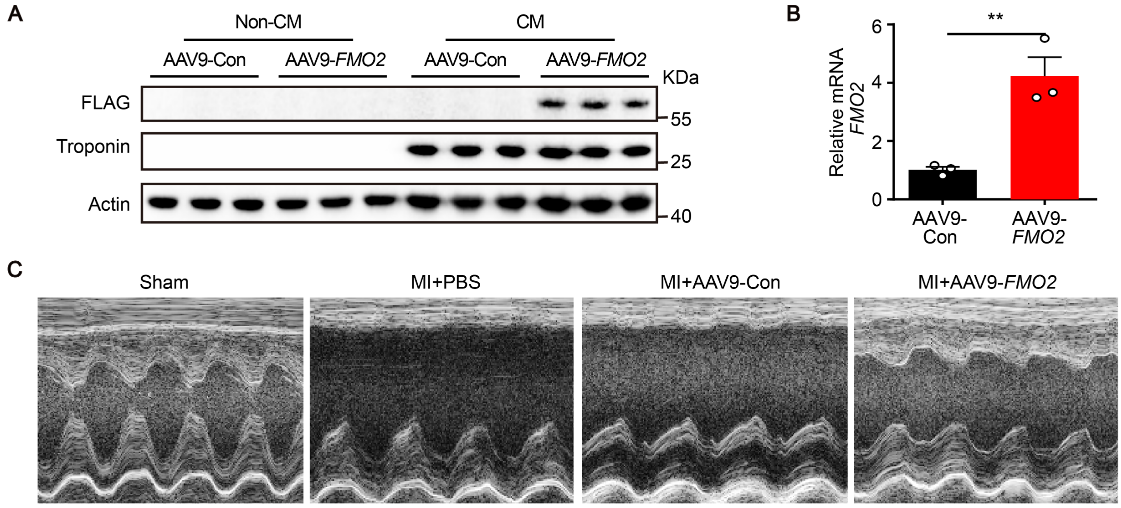

Supplemental Figure 5. Effect of AAV9-FMO2 in rat hearts. (**A**) Expression of FMO2-FLAG and Troponin in CMs and Non-CMs following AAV9-FMO2 injection. (**B**) FMO2 mRNA level in CMs isolated from control and AAV9-FMO2 rat hearts (n = 3 per group). (**C**) Representative photographs of M-mode echocardiography. Quantified data are presented as means ± SEM. Comparisons between two groups were assessed via the Student's t-test. ***p*<0.01.

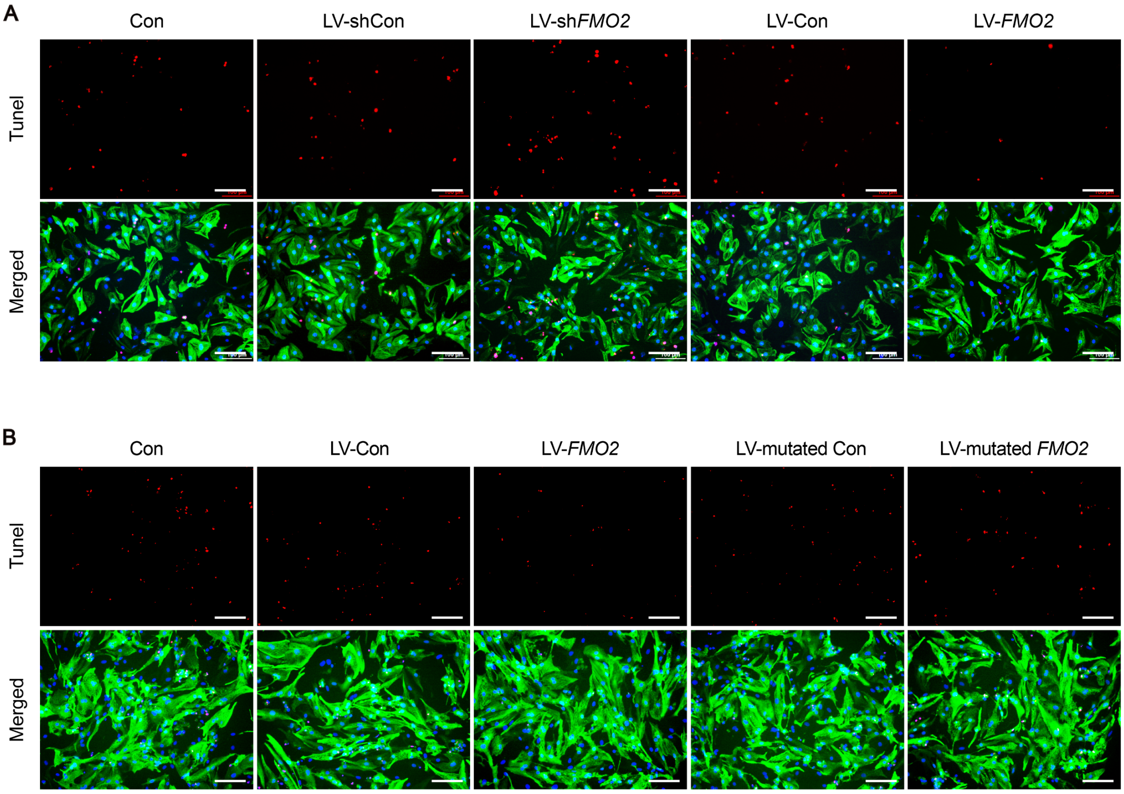

Supplemental Figure 6. FMO2 confers protection against hypoxia-induced apoptosis in cultured cardiomyocytes. (**A**) NRCMs were transfected with either LV-sh*FMO2* or LV-*FMO2* lentivirus, then subjected to hypoxia for 24 hours. Representative views of TUNEL-positive cardiomyocytes are shown. Bar = 100 um. (**B**) TUNEL staining of hypoxia-exposed NRCMs with LV-*FMO2* or enzyme-inactivated *FMO2* (LV-mutated *FMO2*). Bar = 100 um.

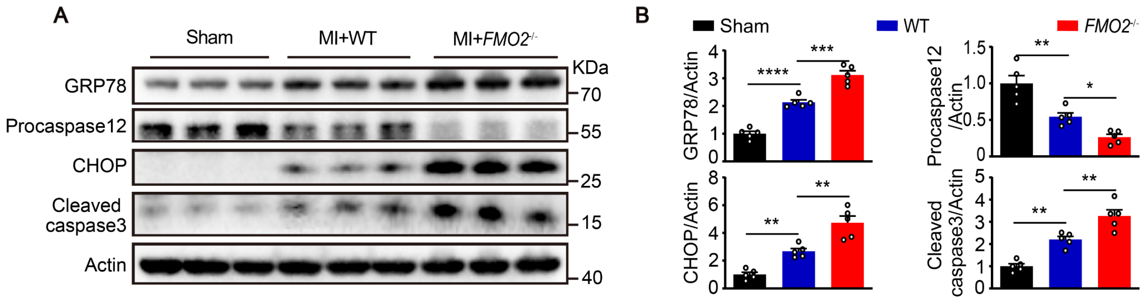

Supplemental Figure 7. Knockout of FMO2 exacerbates ER stress in infarcted hearts. (**A** and **B**) Protein expression of GRP78 and ER stress-induced apoptotic proteins in infarcted hearts of *FMO2*$^{-/-}$ rats, compared with WT. The graphs summarize data from 5 rats per group. Quantified data are presented as means ± SEM, and significance was evaluated via one-way ANOVA with Tukey test among three or more groups. *$p<0.05$, **$p<0.01$, ***$p<0.001$, ****$p<0.0001$.

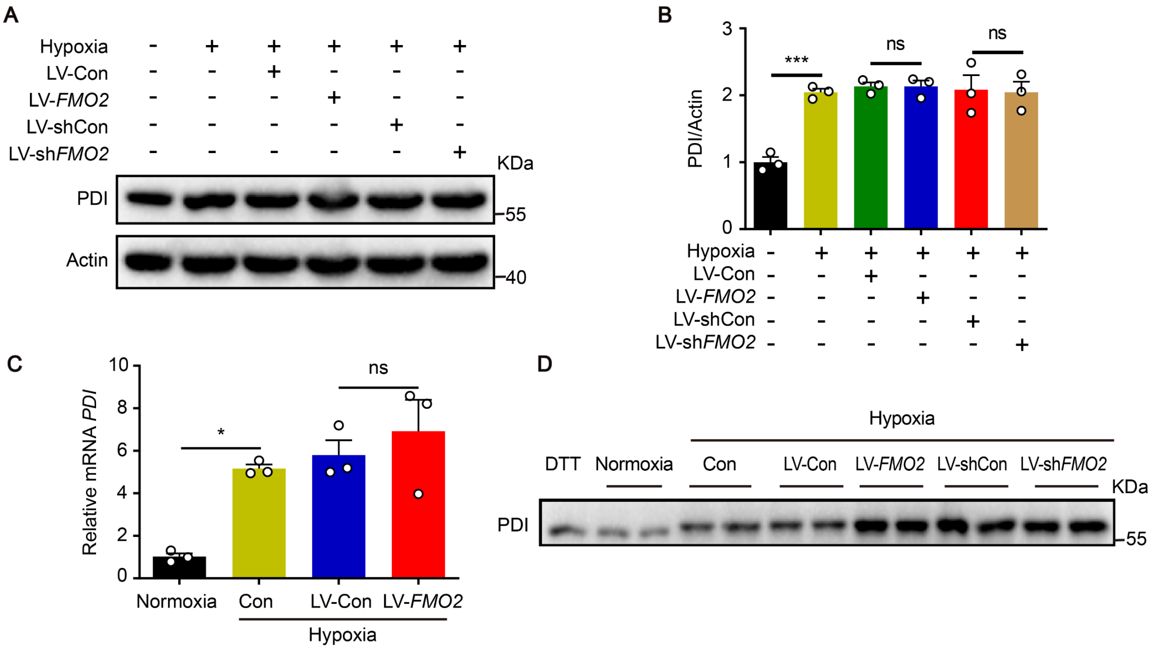

Supplemental Figure 8. Effect of FMO2 on disulfide-bond modification is independent on PDI. (**A-C**) Effect of FMO2 on PDI protein and mRNA levels in NRCMs. (**D**) The redox status of PDI in NRCMs transfected with LV-shFMO2 or LV-FMO2. DTT was used as reduced control. The graphs summarize data from 3 independent experiments. Quantified data are presented as means ± SEM, and significance was evaluated via one-way ANOVA with Tukey test among three or more groups. **p*<0.05, ****p*<0.001, ns: not significant.

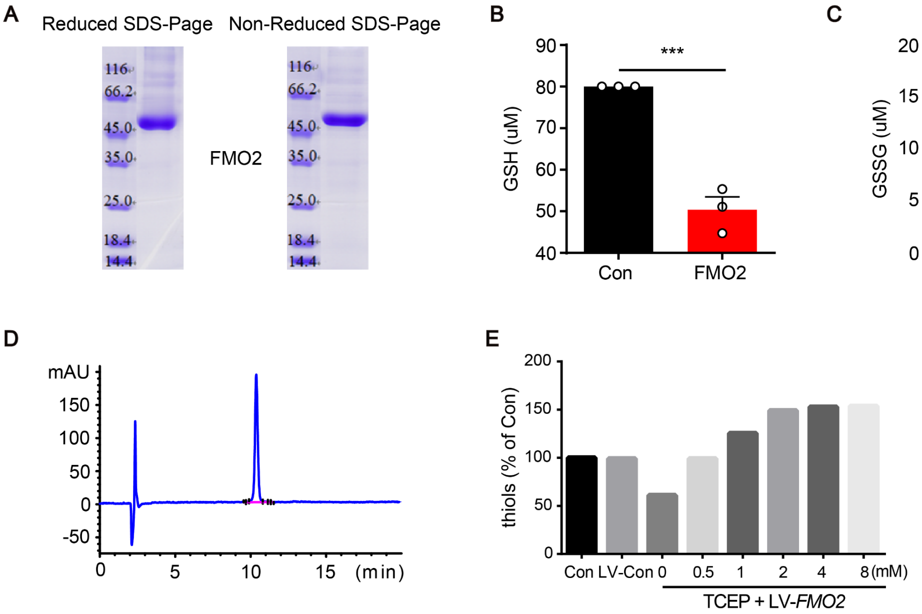

Supplemental Figure 9. FMO2 catalyzes disulfide-bond formation. (**A**) FMO2 protein was purified from insect cells and estimated on the Coomassie Blue-stained SDS-PAGE gel under reduced and non-reduced conditions. (**B** and **C**) Contents of GSH and GSSG were measured after reaction with or without FMO2. (**D**) Purity of peptide NRCSQGSCWN was analyzed by High Performance Liquid Chromatography (HPLC). (**E**) TCEP inhibited disulfide-bond formation in a dose-dependent manner. Bar diagram of differently treated groups was normalized to the hypoxia control. Quantified data are presented as means ± SEM, and significance was evaluated via one-way ANOVA with Tukey test among three or more groups. ***p<0.001.

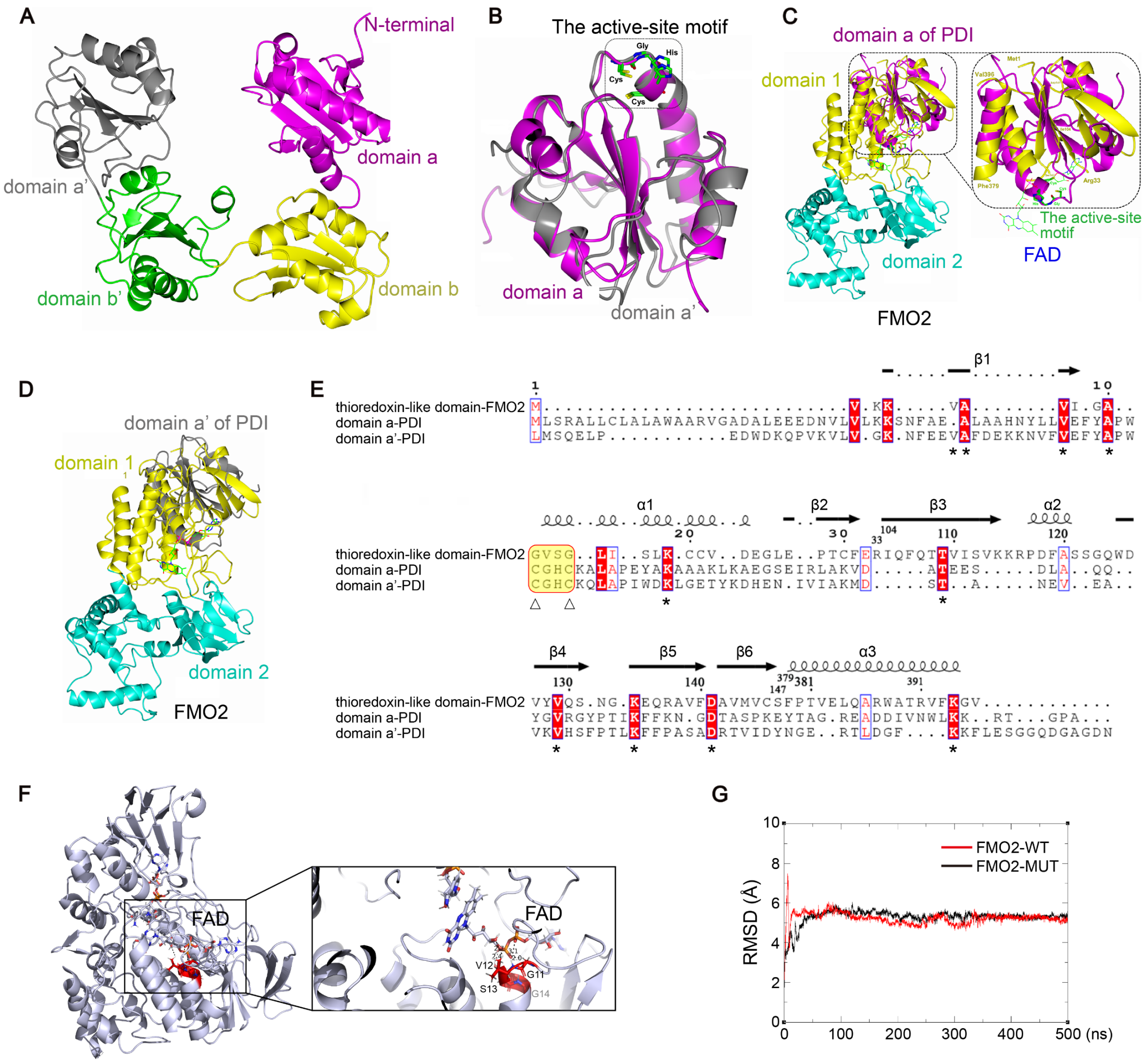

Supplemental Figure 10. Structures of PDI and FMO2. (**A** and **B**) Overall structure of PDI. (**A**) N-terminal, domain a, b, b' and a' are colored in magenta, yellow, green and grey, respectively. (**B**) The active-site motif in domain a and a' (colored in magenta and grey). (**C**) Ribbon diagram of FMO2 (domain 1 and domain 2 colored in yellow and cyan, respectively) was superposed with that of PDI domain a (colored in magenta). Close-up view of the active site is shown in right. (**D**) Ribbon diagram of FMO2 (domain 1 and 2 colored in yellow and

cyan, respectively) was superposed with that of PDI domain a’ (colored in magenta). (**E**) Structure-based sequence alignment of the thioredoxin-like domain from FMO2 and domains a and a’ from PDI. Arrows represent β-strands and helices represent α-helices. The active-site motif CGHC is colored in yellow and the conserved residues are colored in red and marked by *. (**F**) The GVSG motif of FMO2 anchors FAD through hydrogen bonding. The amino acids in the GVSG motif are highlighted in red. The hydrogen bonding interaction between GVSG and FAD is depicted by black dotted lines. (**G**) Molecular dynamics simulations for FMO2-WT and FMO2-MUT were conducted. Both FMO2-WT and FMO2-MUT were subjected to a 500 ns molecular dynamics simulation, and the RMSD (Å) was calculated during the simulation.

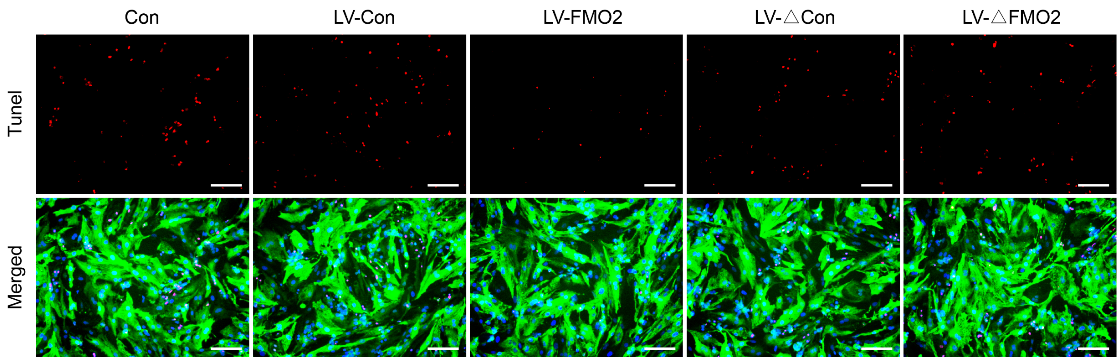

Supplemental Figure 11. GVSG-mutated FMO2 fails to reduce cardiomyocyte apoptosis.

TUNEL staining of hypoxia-exposed NRCMs with LV-*FMO2* or LV-△*FMO2*. Bar = 100 um.

△FMO2 indicates GVSG-mutant FMO2.

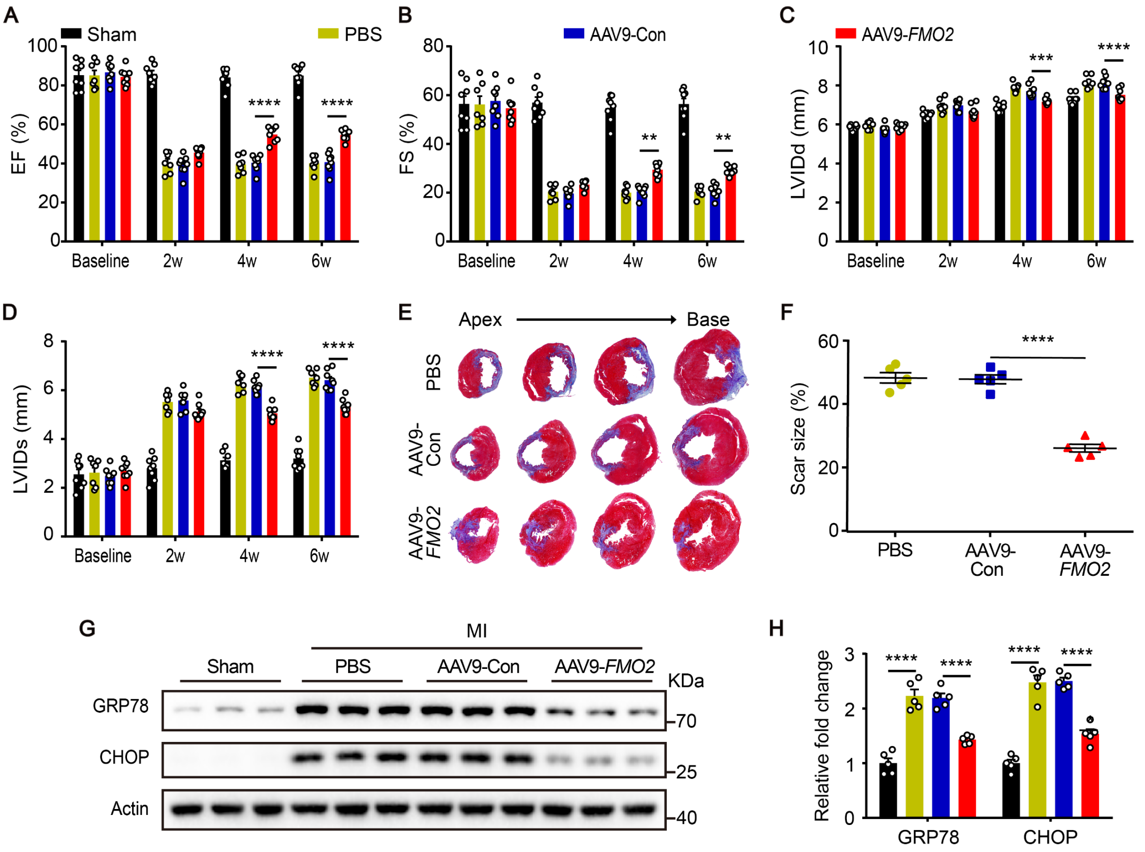

Supplemental Figure 12. AAV9-mediated overexpression of FMO2 protects the heart from myocardial infarction. (**A-D**) Quantitative analysis of echocardiography (n = 7-8 per group). (**E** and **F**) Representative tissue sections stained with Masson at 28 days after MI injury. Percentage of scar size in is shown in (right, n = 5 per group). (**G** and **H**) Protein expression of ER stress markers in rats that either received AAV9-*FMO2* or AAV9-Con virus. Protein lysates were harvested from infarct border zone of hearts (n = 5 per group). Quantified data are presented as means ± SEM. Comparisons among three or more groups were evaluated via one-way analysis of variance (ANOVA) with Tukey test, and comparisons among groups after

multiple treatments were evaluated via two-way ANOVA with Tukey test. ** $p<0.01$, *** $p<0.001$, **** $p<0.0001$.

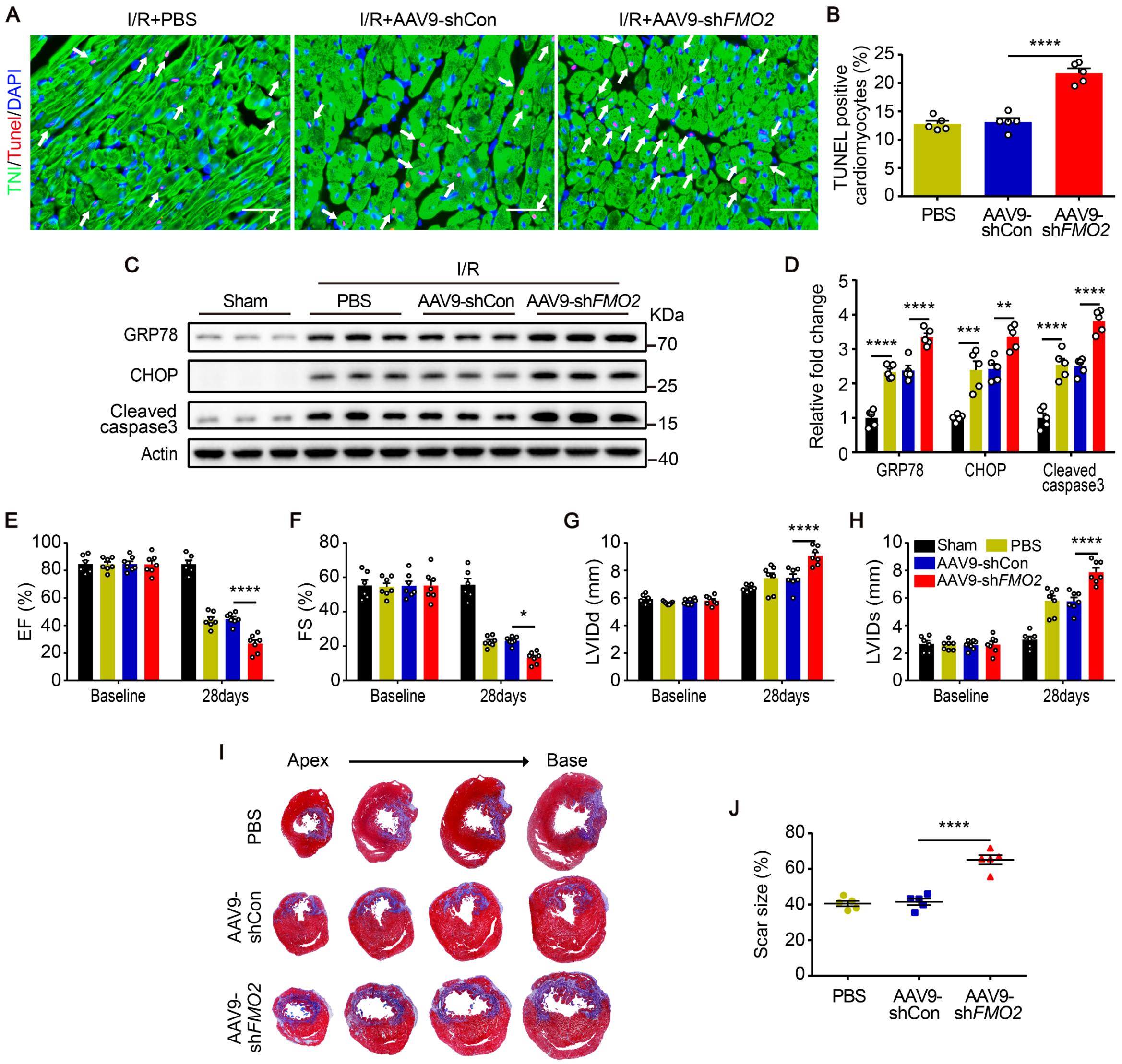

Supplemental Figure 13. Cardiomyocyte-specific FMO2 knockdown impairs cardiac function accompanied by increased cardiomyocyte apoptotic level after I/R. (**A** and **B**) Representative TUNEL staining images of rat heart sections and quantitative results after I/R surgery (n = 5 per group). Bar = 50 μm. (**C** and **D**) Protein expression of GRP78 and ER stress-induced apoptotic proteins in rats that either received AAV9-sh*FMO2* or AAV9-shCon virus. Protein lysates were harvested from infarct border zone of hearts (n = 5 per group). (**E-H**)

Quantitative analysis of echocardiography (n = 6 in Sham group, n = 7 in other groups). (**I** and **J**) Representative tissue sections stained with Masson at 28 days post-I/R. Percentage of scar size in is shown in (right, n = 5 per group). Quantified data are presented as means ± SEM. Comparisons among three or more groups were evaluated via one-way analysis of variance (ANOVA) with Tukey test, and comparisons among groups after multiple treatments were evaluated via two-way ANOVA with Tukey test. *$p<0.05$, **$p<0.01$, ***$p<0.001$, ****$p<0.0001$.

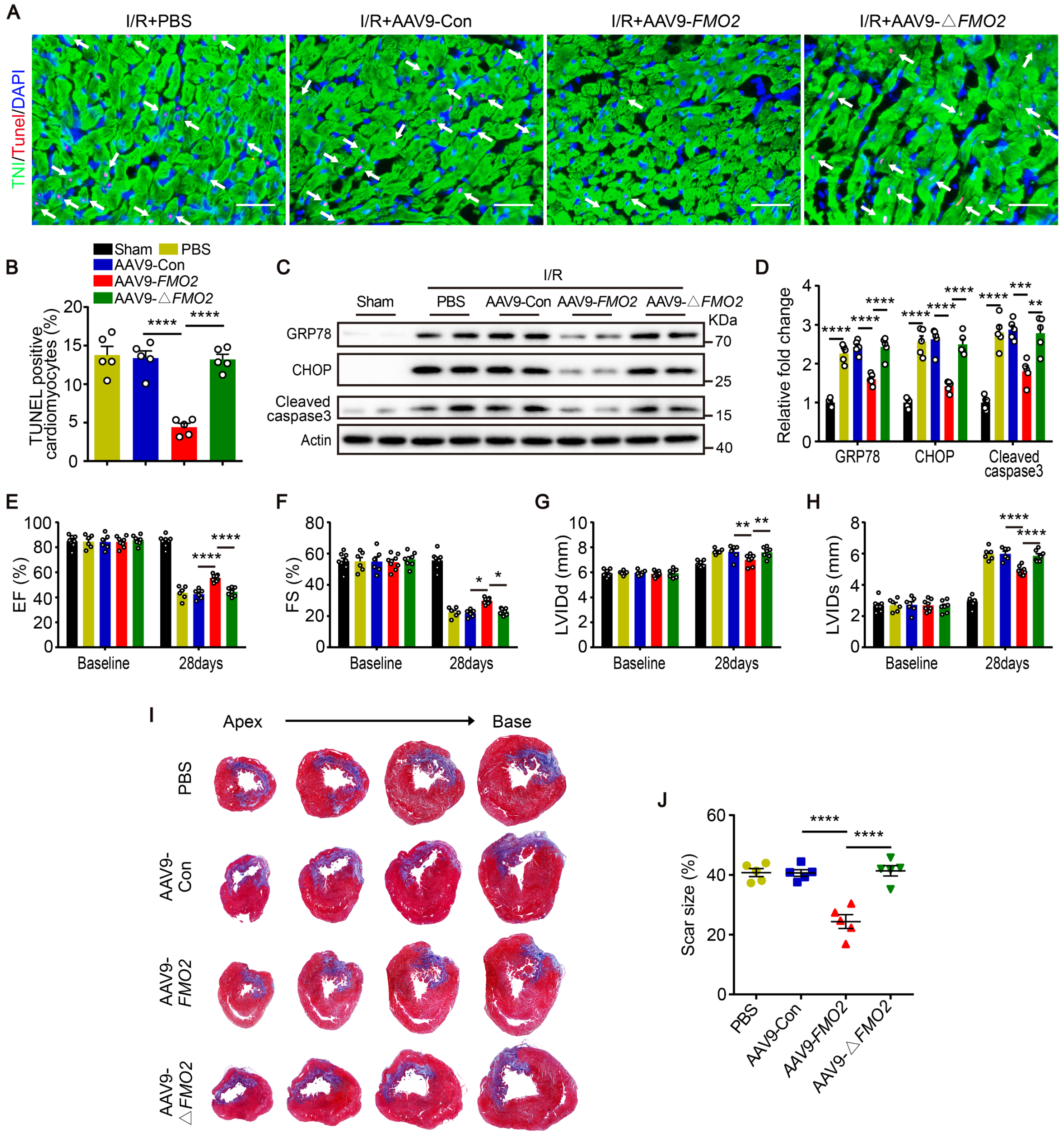

Supplemental Figure 14. Cardiomyocyte-specific FMO2 overexpression decreases cardiomyocyte apoptosis and improves cardiac function after I/R. (**A** and **B**) Representative sections of rat hearts subjected to I/R were analyzed for apoptosis by TUNEL staining. Bar = 50 μm. Quantitative analysis of TUNEL-Troponin I positive cardiomyocytes is

shown in (right, n = 5 per group). (**C** and **D**) Protein expression of GRP78 and ER stress-induced apoptotic proteins in rats that either received AAV9-*FMO2* or AAV9-△*FMO2* virus. Protein lysates were harvested from infarct border zone of hearts (n = 5 per group). (**E-H**) Quantitative analysis of echocardiography (n = 6-8 per group). (**I** and **J**) Representative tissue sections stained with Masson at 28 days after I/R injury. Percentage of scar size in is shown in (right, n = 5 per group). Quantified data are presented as means ± SEM. Comparisons among three or more groups were evaluated via one-way analysis of variance (ANOVA) with Tukey test, and comparisons among groups after multiple treatments were evaluated via two-way ANOVA with Tukey test. *$p<0.05$, **$p<0.01$, ***$p<0.001$, ****$p<0.0001$. △ FMO2 indicates GVSG-mutant FMO2.

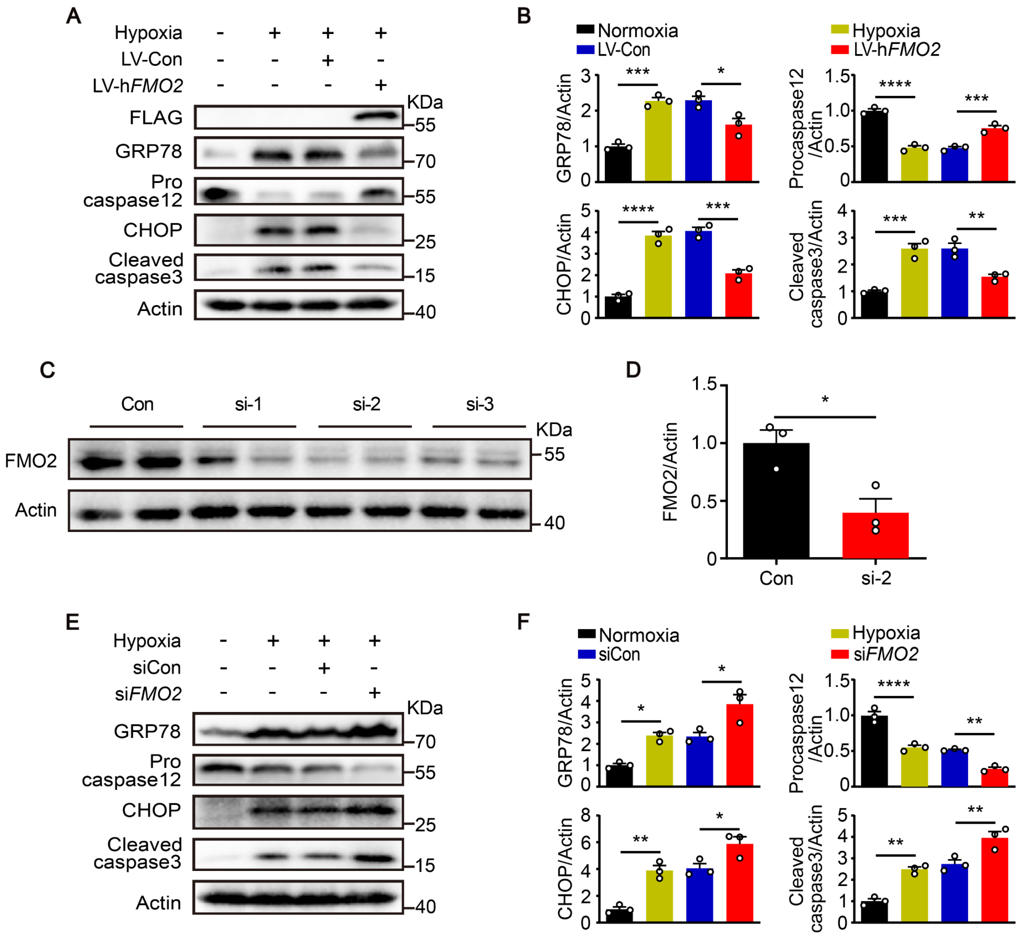

Supplemental Figure 15. FMO2 inhibits ER stress and ER stress-induced apoptotic response in hiPSC-CMs. (**A** and **B**) Protein expression of GRP78 and ER stress-induced apoptotic proteins in hypoxia-exposed hiPSC-CMs transfected with LV-h*FMO2* lentivirus. (**C** and **D**) FMO2 protein expression in hiPSC-CMs after siRNA (siFMO2) transfection. si-2 siRNA targeting human FMO2 was used in the following experiments. (**E** and **F**) Protein expression of GRP78 and ER stress-induced apoptotic proteins in hypoxia-exposed hiPSC-CMs transfected with *FMO2* siRNA. The graphs summarize data from 3 independent

experiments. Quantified data are presented as means ± SEM, and significance was evaluated via one-way ANOVA with Tukey test among three or more groups. *p<0.05, **p<0.01, ***p<0.001, ****p<0.0001, ns: not significant.

SNP	Chr:Position (GRCh38)	Effect allele	Other allele	Exposure: *FMO2* expression in left ventricle			Outcome: HF			MR results				
				Beta	SE	*p*val	Beta	SE	*p*val	Method	Beta	SE	OR (95% CI)	*p*val
rs78893152	1:171167180	A	G	-0.440	0.071	5.4x10$^{\wedge -10}$	0.040	0.019	0.034	Wald ratio	-0.090	0.043	0.914 (0.841-0.993)	0.034

Supplemental Table 1. Causality analysis between FMO2 and heart failure. SE, standard error; CI, confidence interval; OR, odds ratio.
