# Supplementary material for: Flavin-containing monooxygenase 2 confers cardioprotection in ischemia models through its disulfide bond catalytic activity
Source: J Clin Invest. 2024 Oct 31;134(24):e177077. doi: 10.1172/JCI177077 (PMC11645147; doi:10.1172/JCI177077)

## Figure 1D

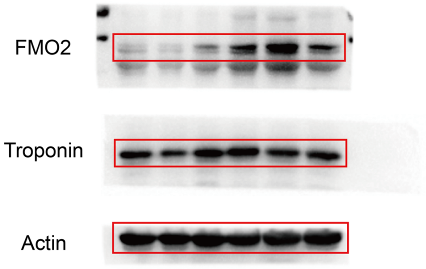


## Figure 1F

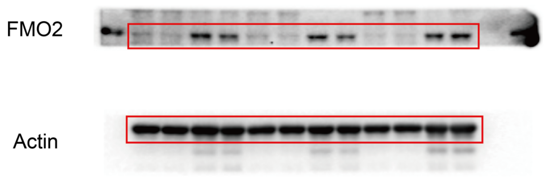


## Figure 2C

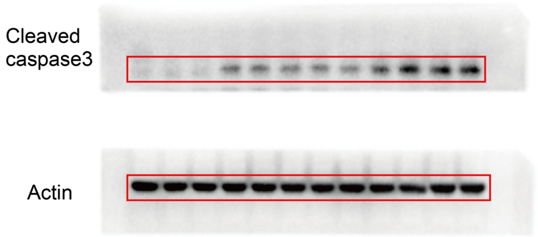


## Figure 3C

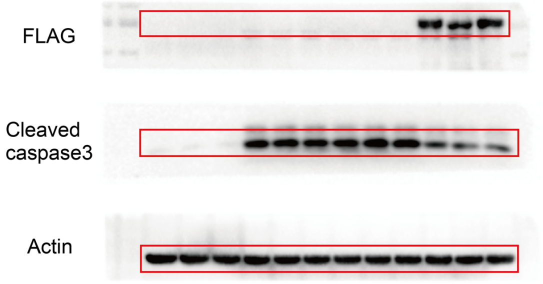

## Figure 4A

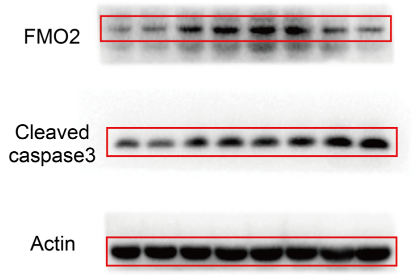


## Figure 4C

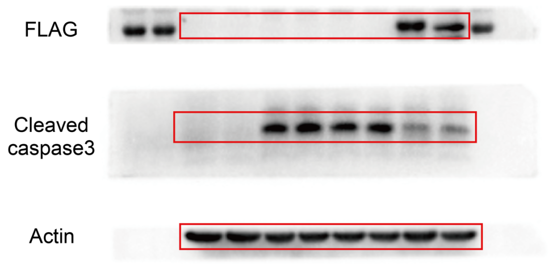


## Figure 4G

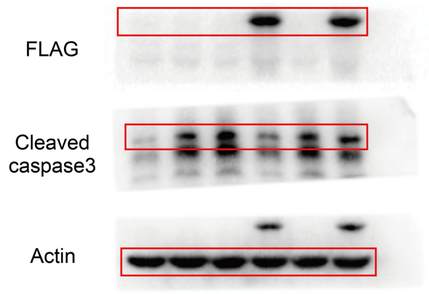

## Figure 5C

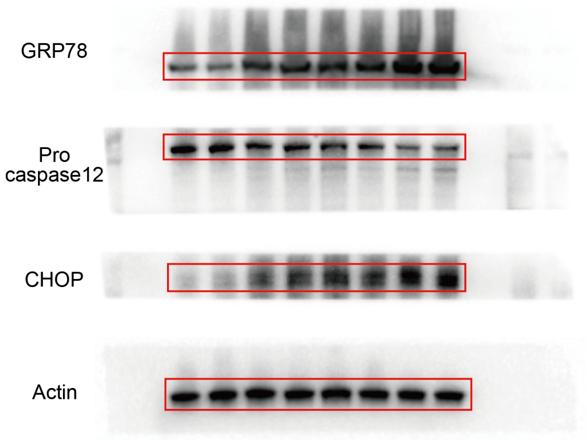


## Figure 5E

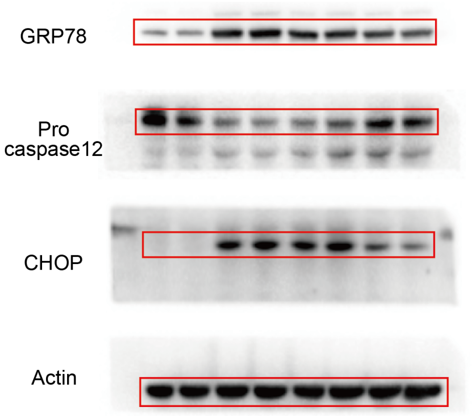


## Figure 5H

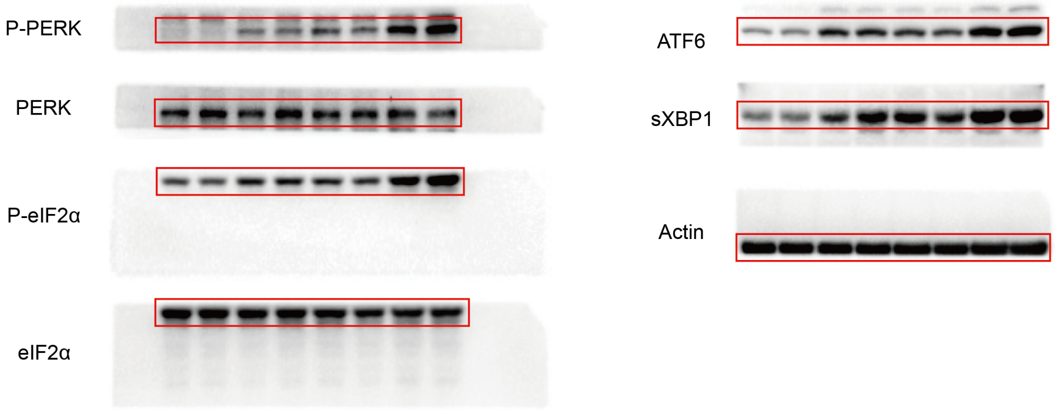

## Figure 5J

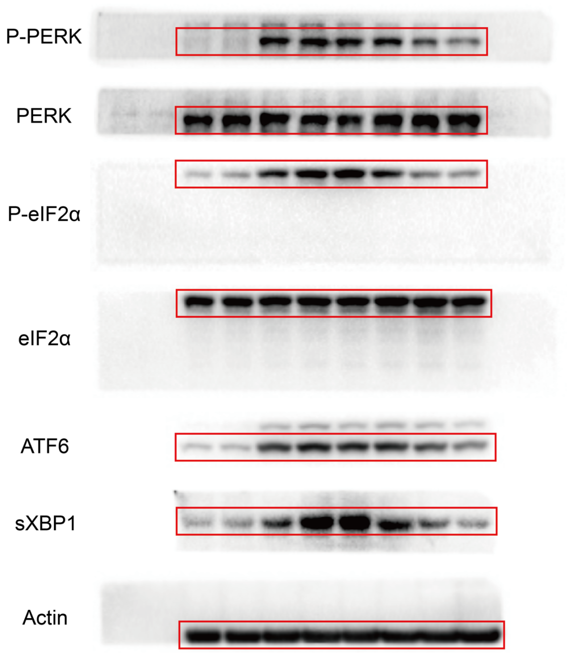


## Figure 5L

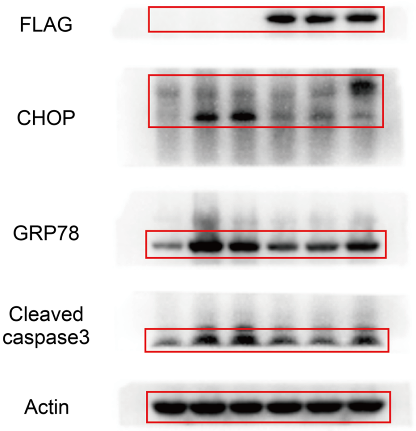

**Figure 6B**

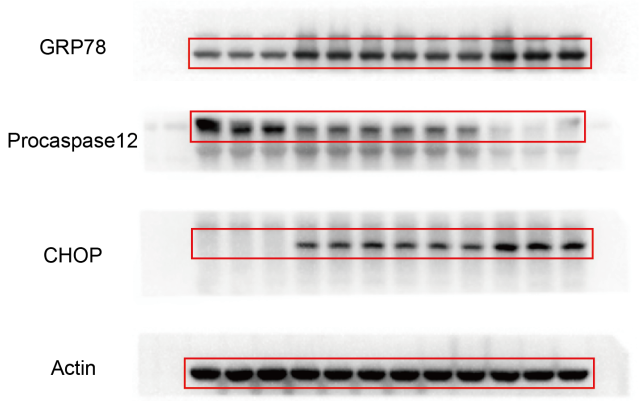


**Figure 6D**

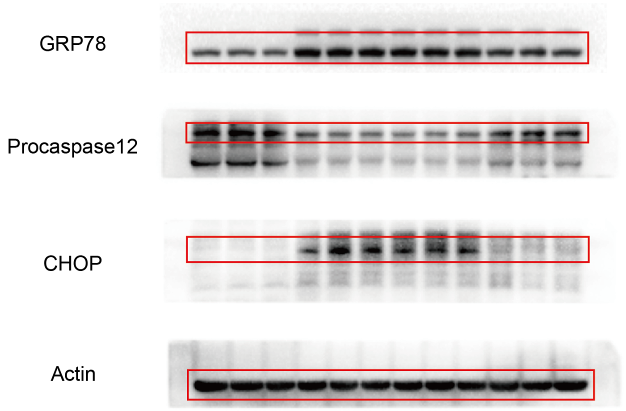

## Figure 6F

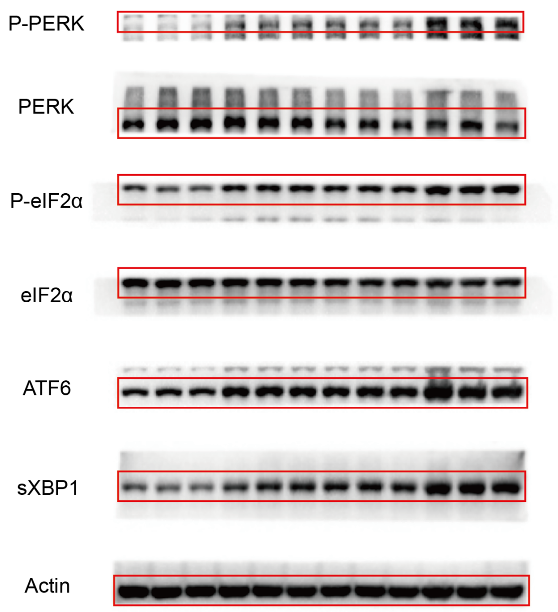


## Figure 6H

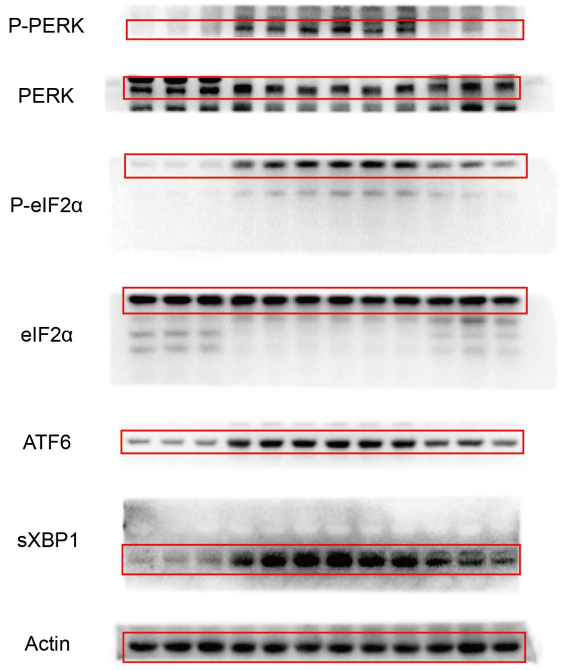

## Figure 7G

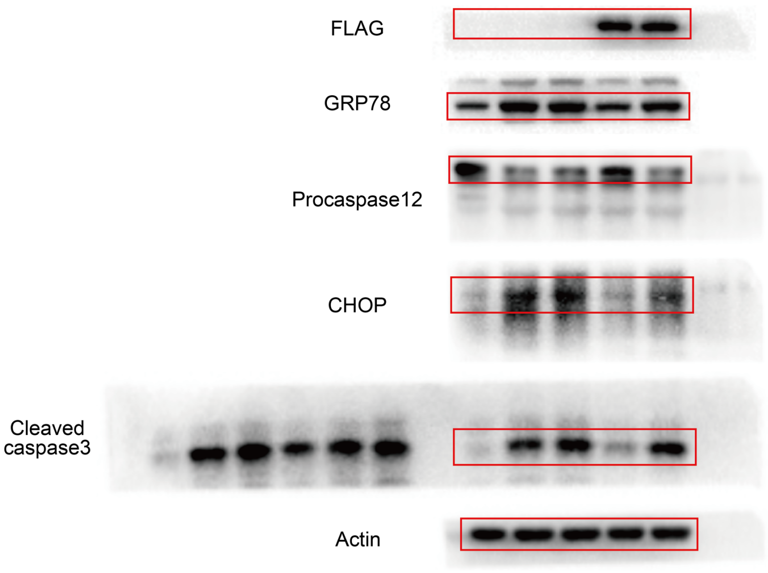


## Figure 7J

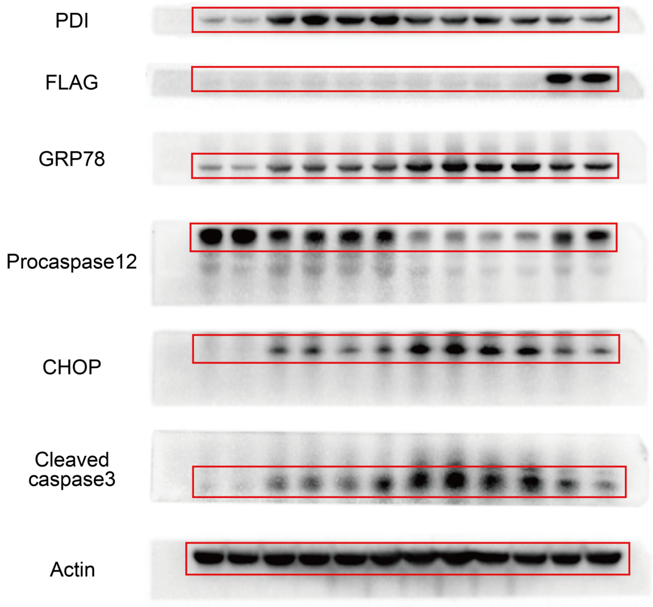

## Figure 8I

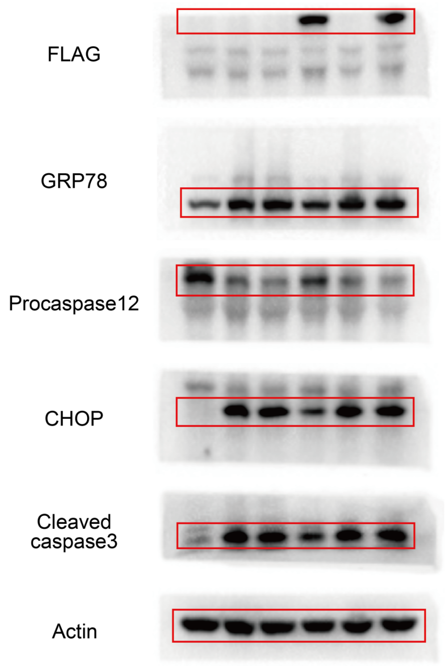

## Supplemental Figure 1C

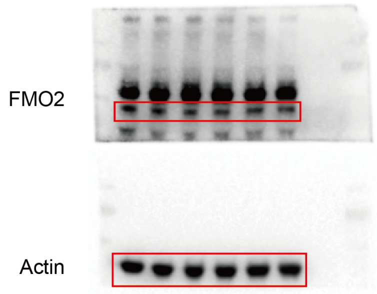


## Supplemental Figure 1E

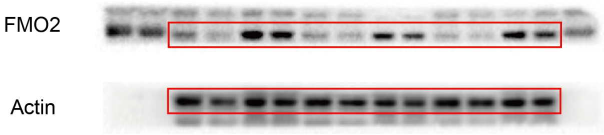


## Supplemental Figure 1G

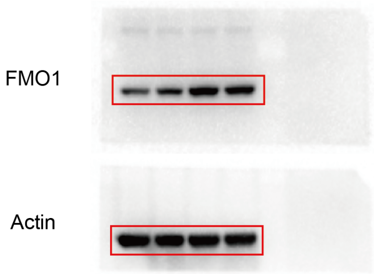


## Supplemental Figure 1I

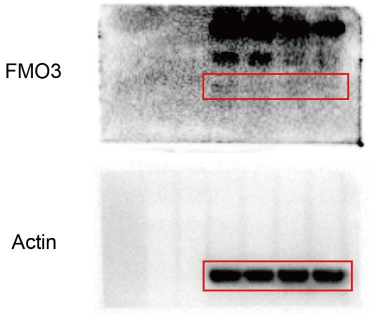


## Supplemental Figure 1K

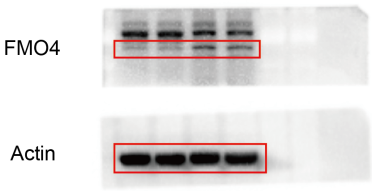


## Supplemental Figure 1M

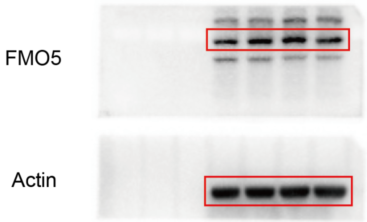

## Supplemental Figure 2D

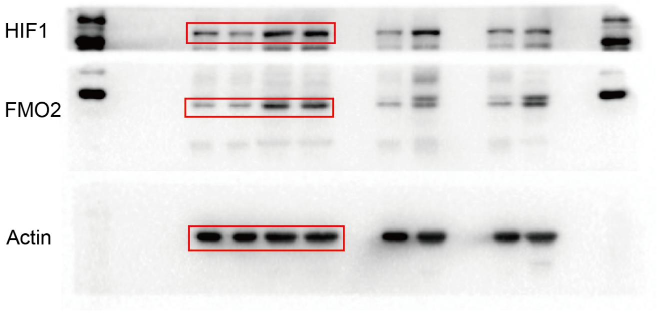


## Supplemental Figure 2F

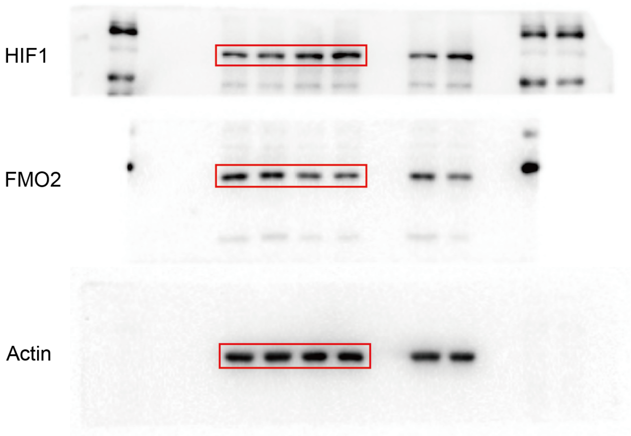

## Supplemental Figure 3A

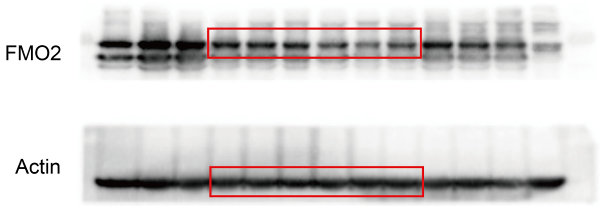


## Supplemental Figure 4C

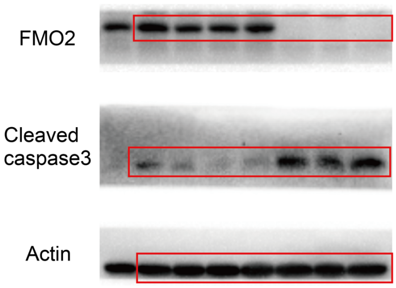

## Supplemental Figure 5A

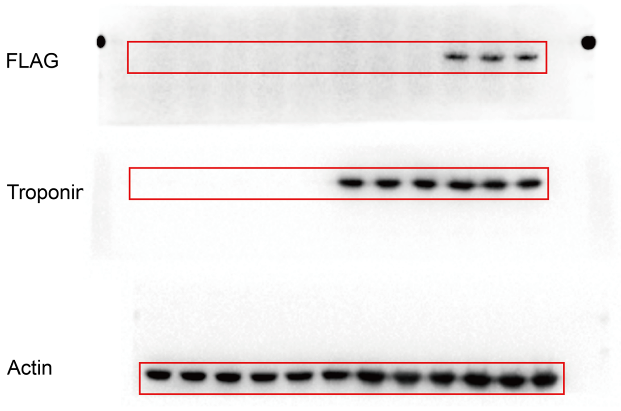


## Supplemental Figure 7A

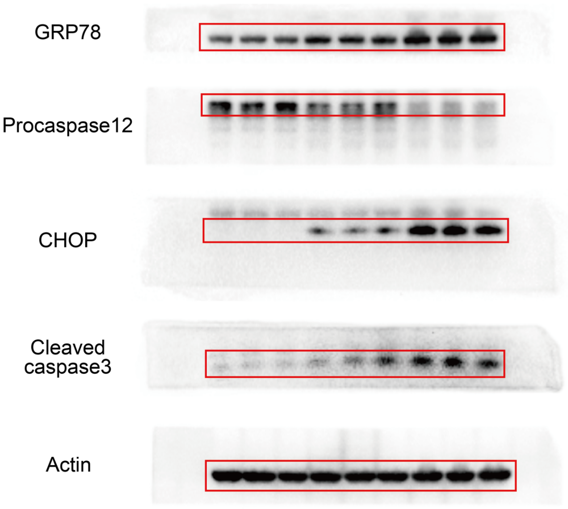

## Supplemental Figure 8A

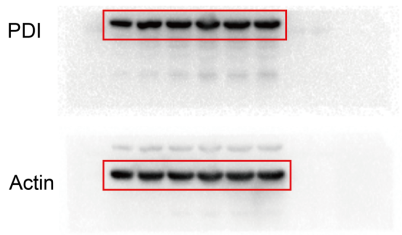


## Supplemental Figure 8D

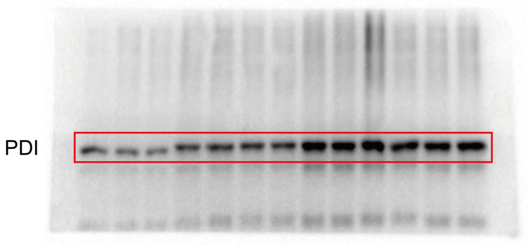


## Supplemental Figure 12G

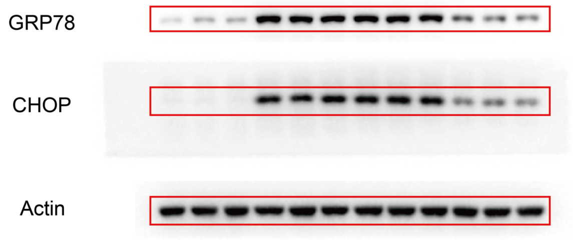


## Supplemental Figure 13C

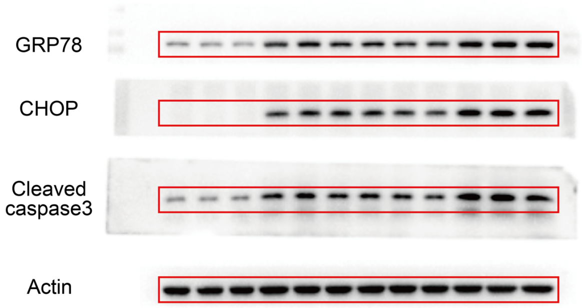

## Supplemental Figure 14C

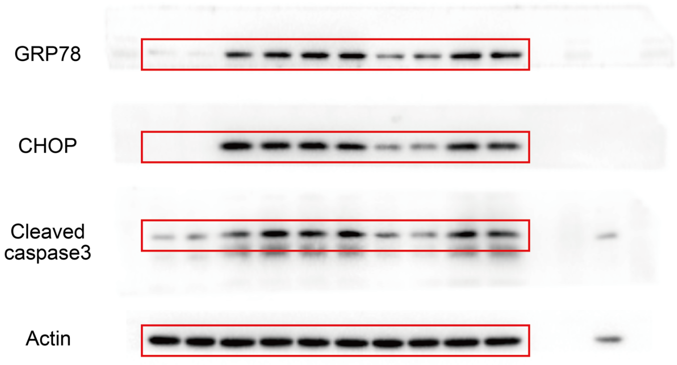


## Supplemental Figure 15A

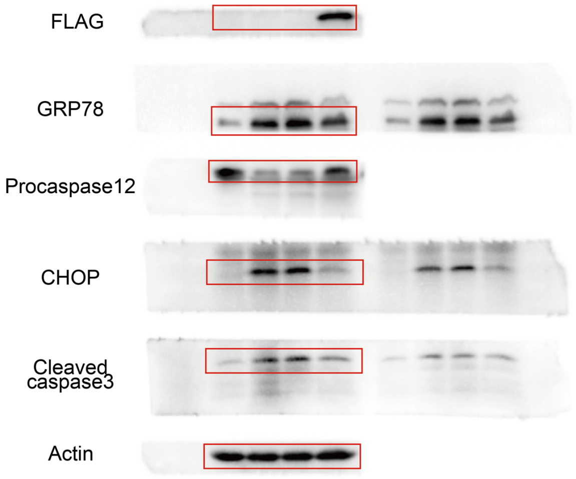

## Supplemental Figure 15C

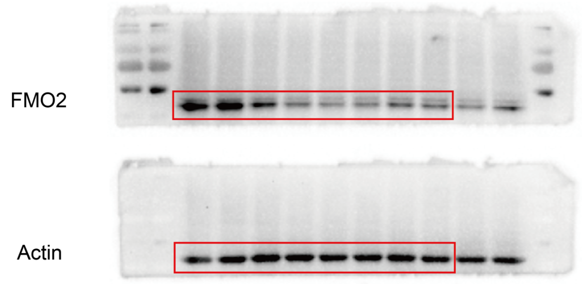


## Supplemental Figure 15E

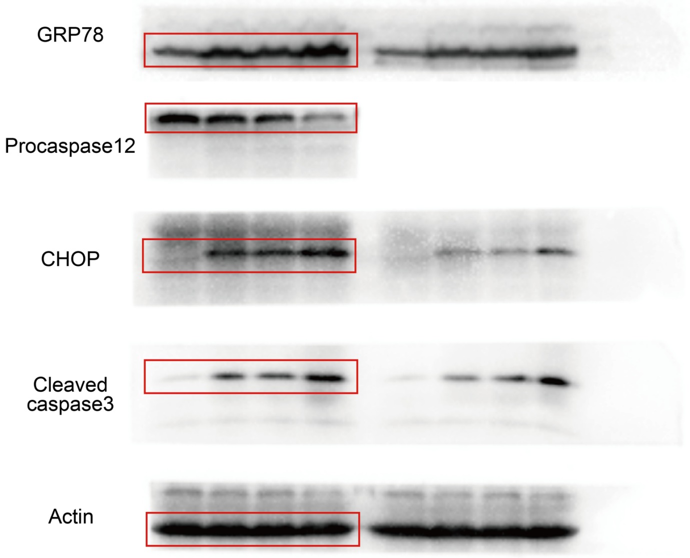

Supplement: Unedited blot and gel images [file jci-134-177077-s223.pdf]
